# HBD-2 variants and SARS-CoV-2: New insights into inter-individual susceptibility

**DOI:** 10.3389/fimmu.2022.1008463

**Published:** 2022-12-09

**Authors:** Mohammed Y. Behairy, Mohamed A. Soltan, Muhammad Alaa Eldeen, Jawaher A. Abdulhakim, Maryam M. Alnoman, Mohamed M. Abdel-Daim, Hassan Otifi, Saleh M. Al-Qahtani, Mohamed Samir A. Zaki, Ghadi Alsharif, Sarah Albogami, Ibrahim Jafri, Eman Fayad, Khaled M. Darwish, Sameh S. Elhady, Refaat A. Eid

**Affiliations:** ^1^ Department of Microbiology and Immunology, Faculty of Pharmacy, University of Sadat City, Sadat City, Egypt; ^2^ Department of Microbiology and immunology, Faculty of Pharmacy, Sinai University – Kantara Branch, Ismailia, Egypt; ^3^ Cell Biology, Histology & Genetics Division, Biology Department, Faculty of Science, Zagazig University, Zagazig, Egypt; ^4^ Medical Laboratory Department, College of Applied Medical Sciences, Taibah University, Yanbu, Saudi Arabia; ^5^ Biology Department, Faculty of Science, Taibah University, Yanbu, Saudi Arabia; ^6^ Department of Pharmaceutical Sciences, Pharmacy Program, Batterjee Medical College, Jeddah, Saudi Arabia; ^7^ Pharmacology Department, Faculty of Veterinary Medicine, Suez Canal University, Ismailia, Egypt; ^8^ Pathology Department, College of Medicine, King Khalid University, Abha, Saudi Arabia; ^9^ Department of Child Health, College of Medicine, King Khalid University, Abha, Saudi Arabia; ^10^ Anatomy Department, College of Medicine, King Khalid University, Abha, Saudi Arabia; ^11^ Department of Histology and Cell Biology, College of Medicine, Zagazig University, Zagazig, Egypt; ^12^ College of Clinical Laboratory Sciences, King Saud bin Abdulaziz University for Health Sciences, Jeddah, Saudi Arabia; ^13^ Department of Biotechnology, College of Sciences, Taif University, Taif, Saudi Arabia; ^14^ Department of Medicinal Chemistry, Faculty of Pharmacy, Suez Canal University, Ismailia, Egypt; ^15^ Department of Natural Products, Faculty of Pharmacy, King Abdulaziz University, Jeddah, Saudi Arabia

**Keywords:** COVID-19, SNPs, molecular dynamics, antimicrobial peptides, *h*BD-2

## Abstract

**Background:**

A deep understanding of the causes of liability to SARS-CoV-2 is essential to develop new diagnostic tests and therapeutics against this serious virus in order to overcome this pandemic completely. In the light of the discovered role of antimicrobial peptides [such as human b-defensin-2 (hBD-2) and cathelicidin LL-37] in the defense against SARS-CoV-2, it became important to identify the damaging missense mutations in the genes of these molecules and study their role in the pathogenesis of COVID-19.

**Methods:**

We conducted a comprehensive analysis with multiple in silico approaches to identify the damaging missense SNPs for hBD-2 and LL-37; moreover, we applied docking methods and molecular dynamics analysis to study the impact of the filtered mutations.

**Results:**

The comprehensive analysis reveals the presence of three damaging SNPs in hBD-2; these SNPs were predicted to decrease the stability of hBD-2 with a damaging impact on hBD-2 structure as well. G51D and C53G mutations were located in highly conserved positions and were associated with differences in the secondary structures of hBD-2. Docking-coupled molecular dynamics simulation analysis revealed compromised binding affinity for hBD-2 SNPs towards the SARS-CoV-2 spike domain. Different protein–protein binding profiles for hBD-2 SNPs, in relation to their native form, were guided through residue-wise levels and differential adopted conformation/orientation.

**Conclusions:**

The presented model paves the way for identifying patients prone to COVID-19 in a way that would guide the personalization of both the diagnostic and management protocols for this serious disease.

## Introduction

The emergence of the novel coronavirus disease 2019 (COVID-19), which impacted global health deleteriously, has attracted worldwide attention in terms of fighting this highly transmissible virus (1). One basic point that is studied continuously with any novel infective agent is the fighting mechanism of the human immune system against this newly emerged pathogen and how this mechanism could affect the transmission and the complications (2). Human antimicrobial peptides (AMPs) have been extensively studied for their roles in fighting against several forms of pathogens such as bacteria and viruses where several trials have been performed to incorporate these peptides with antibiotics for fighting against bacteria (3). Generally, AMPs have demonstrated antiviral activity through different mechanisms where blocking the contact between the virus itself and the human cellular target represented a major mechanism. Human β-defensins (*h*BDs), a leading class of AMPs, were found in several mucosal sites to perform an innate immune defense mechanism against microbial colonization (4). Recently, this potential mechanism was studied specifically for *h*BD-2, which was able to bind to SARS-CoV-2 RBD and inhibit the binding of this RBD to *h*ACE2, leading to the inhibition of the virus spreading, which pointed out the role of this AMP in fighting against COVID-19 (5). In addition, a recent study found that patients who developed severe COVID-19 had low serum levels of *h*BD-2 (6). Human cathelicidin LL‐37 is another example of AMPs that possess antimicrobial activity through the neutralization of the bacterial lipopolysaccharides (7). This AMP was also correlated with COVID-19 where it was found that the deficiency of vitamin D supplementation would negatively affect the level of LL-37 and allow for viral propagation (8). In addition, the high affinity of LL-37 to the RBD of this serious virus and its role in fighting against COVID-19 has been confirmed as well (9, 10).

Single-nucleotide polymorphism (SNP) is a gene-specific site variation that occurs in one base of the DNA nucleotide where it represents the most abundant form of human genetic variation (11). Generally, SNPs can be classified into different forms where missense SNPs, which are characterized by an amino acid substitution, can affect the development of different diseases as well as the human response to that disease progression (12, 13). This amino acid substitution may lead to the generation of a new mutated protein with structural and functional characteristics that largely deviated from the native one. Consequently, the downstream signals and the roles of the newly mutated protein would be significantly different from the original one, an approach that results in a novel way of disease development for cases that experienced that mutation (14, 15). The downstream effect of a specific SNP can range from the modification of a protein solubility or stability to the deregulation of transcription controlling proteins, which, in turn, affects the protein expression machinery in the cell (16).

Examples of the deleterious outcome of missense SNPs include the effects of SNPs in defensin genes on the liability to human immunodeficiency virus (HIV) infection (17), the effect of KRAS SNP on cell division and tumor progression (18), the role of rs4986790 SNP in the toll-like receptor 4 (TLR4) gene in patients’ vulnerability to HIV-1 infection (19), and the impact of AGER SNPs on diabetes complications and heart diseases (20). It is noteworthy that the continuous development in sequencing technology contributed largely to the elevation in the number of reported SNPs for many studied genes. While that development is advantageous, it became essential to study the implications of those reported SNPs and filter out the pathogenic ones from normal variants (21). As mentioned, the large number of reported SNPs makes it difficult to analyze through wet lab experiments and clinical studies, and alternatively, bioinformatics tools, which witnessed a great revolution in the last few years, make it feasible to access and filter a large number of reported SNPs and nominate the most deleterious ones in a timely and cost-effective method (22, 23). These *in silico* methods were widely used in recent years in various immunological and medical aspects (24–26). Moreover, the development in the structural biology field provides an effective way for altered protein structural prediction where the consequences of these structural modifications in the mutated protein can be assessed through molecular docking and molecular dynamics simulation approaches (27).

Hence, the current work aims to study the correlation between the deleterious SNPs of two AMPs, namely, *h*BD-2 and LL-37, and the propagation of COVID-19. We planned to retrieve the missense SNPs of these AMPs and nominate the most deleterious ones after a filtration process. Additionally, we investigated the functional and structural consequences of these SNPs and studied the correlation with the spread of our target virus, SARS-CoV-2. Altogether, these data would shed light on the mechanisms of the inter-individual susceptibility to this serious viral disease and contribute significantly to the field of personalized medicine in the fight against this deadly virus.

## Materials and methods

### General information

The retrieval of general information related to defensin beta 4A gene (*DEFB4A)* and cathelicidin antimicrobial peptide gene (*CAMP)* was performed using the National Center for Biotechnology Information (NCBI) database as well as the Ensembl database. Furthermore, Genecards.org’s database was utilized to retrieve information about gene ontology with compartments.jensenlab.org serving as the source for the data on the subcellular localization.

### The retrieval of *DEFB4A* and *CAMP* gene variants

The retrieval of *DEFB4A* gene SNPs and *CAMP* gene SNPs was performed using NCBI databases depending on the variation viewer (https://www.ncbi.nlm.nih.gov/variation/view/) with the selection of dbSNP to be our source database. “DEFB4A” or 1673 [geneid] was the used entry with the *DEFB4A* gene. “CAMP” or 820 [geneid] was the used entry with the *CAMP* gene. The filtration of the retrieved variants was performed and the missense SNPs were solely chosen for the additional analysis.

### Predicting the SNPs with the most deleterious impacts

Six various *in silico* tools were utilized to predict which SNPs have the most deleterious impact on protein function for both *h*BD-2 and LL-37. (1) SIFT (Sorting Intolerant from Tolerant) employs the sequence homology as well as the physical characteristics of amino acids to forecast how variations may affect protein function (https://sift.bii.a-star.edu.sg/) (28). (2) Polymorphism Phenotyping-2 (PolyPhen-2) assesses the effects of substituting amino acids depending on physical approaches as well as comparative techniques (http://genetics.bwh.harvard.edu/pph2) (29). (3) PROVEAN utilizes blast hits and generates the necessary score and prediction (http://provean.jcvi.org/seq_submit.php) (30). (4) SNPs&GO utilizes a functional annotation approach to determine the SNPs with harmful impacts (https://snps.biofold.org/snpsand-go/snps-and-go.html) (31). (5) PHD-SNP utilizes support vector machines (SVMs) to forecast how the novel phenotype resulting from the missense mutations is associated with human illnesses (http://snps.biofold.org/phd-snp/phd-snp.html) (32). (6) SNAP2 was also used with its distinct neural network’s capacity to distinguish between effect SNPs and the other neutral ones (https://rostlab.org/services/snap/) (33). These mutations, which were found to be deleterious by at least five tools, were designated as the most harmful ones. By combining these distinct tools with various methodologies and algorithms, we aimed at improving the precision of our investigation.

### The analysis of the variants’ effects on the stability of the protein

I-Mutant 2.0 as well as Mu-Pro tools were utilized to investigate how the selected mutations affected the stability of the protein. I-Mutant 2.0 employs a support vector machine to forecast the direction and the magnitude of the change in the free energy (DDG) (https://folding.biofold.org/i-mutant/i-mutant2.0.html) (34). The ProTherm database, with its extensive experimental data on the change in free energy associated with the stability of proteins affected by the mutations, was utilized for testing I-Mutant 2.0 (35). Mu-Pro employs a robust support vector machine approach showing 84% accuracy when applied to the cross-validation and the verification process (http://mupro.proteomics.ics.uci.edu/) (36).

### The recognition of the location of the SNPs on protein domains

InterPro was utilized to find the sites of the deleterious mutations on protein domains (https://www.ebi.ac.uk/interpro/). The functional analysis performed by InterPro could reveal the important domains and the key motifs of the chosen protein (37).

### The investigation of the phylogenetic conservation of protein residues

Utilizing ConSurf, the phylogenetic conservation of the protein residues was examined (https://consurf.tau.ac.il). By analyzing the homologous sequences for the existing phylogenetic connections, ConSurf could examine the sequences of the designated protein for phylogenetic conservation (38, 39).

### Secondary structure analysis

PSIPRED tool (http://bioinf.cs.ucl.ac.uk/psipred/) was utilized to analyze the secondary structure of the selected protein and determine the specific alignment for the altered amino acids in the examined secondary structure. Furthermore, the secondary structures in case of the damaging mutations were analyzed as well. PSIPRED could analyze the secondary structure related to a certain protein with the use of position-specific matrices produced by PSI-BLAST (40).

### Analyzing the effects of our variants on the protein structure

Utilizing the HOPE bioinformatics server, the 3D structure of the designed protein could be examined (https://www3.cmbi.umcn.nl/hope/). HOPE utilizes numerous sources for gathering the relevant information along with the production of homology models with the help of the YASARA program in order to carry out the needed functions (41).

### Molecular docking studies

Prior to molecular docking simulations, proteins including the native dimeric *h*BD-2 (PDB: 1FD3) as well as the constructed SNP-variant dimers were independently prepared through the removal of any bound ligands, crystalized solvent, as well as ionic metals/salts, in addition to subsequent protein protonation (42–45). Residues of *h*BD-2 were indexed starting from Gly24 to Pro64, since the first 23 amino acids are reported as a signal sequence being removed *via* the proteolytic advent of signal peptidases permitting the release and secretion of *h*BD-2 (46). Crystallized SARS-CoV-2 spike (PDB: 6m0j) was also prepared as described above to be only included within the subsequent molecular dynamics simulations serving as a positive control reference. Docking the native or variant *h*BD-2 dimers on the receptor-binding domain (RBD) of SARS-CoV-2 spike protein was performed using two online docking servers: ClusPro v2.0 (Boston and Brook Universities; https://cluspro.org/) (47–50) and ZDOCK v3.0.2 (Massachusetts University; https://zdock.umassmed.edu/) (51, 52).

Relying on the Fast Fourier Transform correlation protocol, the ClusPro server predicted the *h*BD-2/RBD complex through a multi-stage process including PIPER-based rigid docking, interaction energy-based conformational filtration, pose ranking based on clustering properties, and finally refinement through energy minimization (48, 53). Interaction energy adopted by ClusPro includes energy terms for van der Waals (E_att_ + E_rep_), electrostatic (E_elec_), and pairwise structure-dependent potentials (E_DARS_) resulting from Decoys as reference state, while it lacks entropic energy terms (48, 53, 54). In this regard, it was suggested to utilize cluster ranking, in terms of cluster populations where *h*BD-2 inbound with the SARS-CoV-2 RBD site was utilized for *h*ACE2 association, rather than the obtained ClusPro interaction energies in order to rank and identify the best-clustered structure complex (5). Concerning docking with ZDOCK, the protocol was more specific since docking constraints were applied to define the spike RBD binding site with the residue range (Ser436-Tyr508) as it depicted high solvent accessibility and reported enrollment within the RBD/*h*ACE2 complex association (5, 44, 55, 56).

### Docking pose assessment and interface analysis

Evaluation of the best docking pose for each bound spike RBD/*h*BD-2 complex obtained from each docking server was proceeded through macromolecular interface analysis using the online PDBePISA v1.5.2 server tool (European Bioinformatics Institute/EMBL-EBI; https://www.ebi.ac.uk/msd-srv/prot_int/cgi-bin/piserver) (57, 58). This tool provided descriptions for the sole and bound protein interface such as interface residues, total solvent-accessible surface area (Å^2^), numbers/types of binding interactions, and the gained solvation free energy (Δ^i^G; kcal/mol), and its *p*-value (Δ^i^G *p*-value) (59). The last two descriptors are indices for higher interface hydrophobicity/protein affinity (high negative Δ^i^G values) and to how far would the protein–protein interface be interaction-specific (*p* < 0.5) (57).

Additional protein–protein interface analysis was performed using the Network Analysis of Protein Structures server (NAPS; International Institute of Information Technology, Hyderabad, India; https://bioinf.iiit.ac.in/NAPS/). This server adopts the network global analysis tool for representing the nodes and backbone edges, in addition to residue–residue contact plots between the *h*BD-2 and spike RBD monomeric units (60). Moreover, an estimation of the spike RBD/*h*BD-2 binding affinity for the predicted docked complex was performed using the Molecular Mechanics energy-guided Generalized Born and Surface Area (MM/GBSA; kcal/mol) calculations that are implemented at the HawkDock server (Zhejiang University; http://cadd.zju.edu.cn/hawkdock/) (61). The HawkDock MM/GBSA calculation permits an estimation of the energy term components including van der Waal, electrostatic, and polar solvation, in addition to the dissection of these energy terms down to the protein’s per-residue energy contributions (62, 63).

### All-atom molecular dynamics simulations

Best docked spike RBD-associated complexes, for the native *h*BD-2 and each SNP variant, were subjected to 100 ns all-atom molecular dynamics simulations under CHARMM36m forcefield and using the GROMACS program (64). Protein complexes were solvated at the TIP3P cubic box under periodic boundary conditions, while maintaining the 10-Å minimum distance between the protein atoms and box boundaries. The net charge of the system was neutralized *via* sufficient 0.15 M sodium and chloride ions. Systems were subjected to the steepest-descent minimization for 0.05 ns, followed by two-staged equilibration at standard thermo- and barostats (Berendsen-temp for NVT ensembles, 1 ns at 310 K, followed by the Parrinello–Rahmann barostat for the NPT ensemble, 1 ns at 1 atm and 310 K) (43, 64). Molecular dynamics were run for 100 ns under the NPT ensemble and Particle-Mesh-Ewald algorithms for computing long-range electrostatic interaction (65). For comparison, the prepared spike RBD/ACE2 complex was used in the reference simulation run adopting the same conditions being detailed.

Trajectory analysis was performed using root-mean-square deviations (RMSDs; Å), the radius of gyration (Rg; Å), RMS-Fluctuations (RMSFs; Å), and solvent-accessible surface area (SASA; nm^2^) *via* the GROMACS in-house analysis scripts relying on the protein’s backbone alpha-carbon atoms. Visual Molecular Dynamics (VMD) software (Illinois University, v1.9.3, Urbana-Champaign, United States) was used for hydrogen bond analysis defining the hydrogen bond distance/angle cutoffs at 3.0 Å and 20°, respectively, and representing the time of formed particular hydrogen bond as % occupancy. Conformational analysis and visualization of the simulated complexes at specified timeframes were done using PyMOL software (Schrödinger; v2.0.6, United States).

### The analysis of gene–gene interactions

By utilizing GeneMANIA, the network describing gene–gene interactions was produced (http://www.genemania.org). GeneMANIA could forecast the genes that have a strong interaction with a selected gene using a variety of resources and types of data (66).

## Results

### General information

Both *DEFB4A* and *CAMP* genes are protein-coding genes with NCBI Gene IDs of 1673 and 820, respectively. The *DEFB4A* gene is located at 8p23.1; it has two exons and a length of 2,040 nucleotides. There is one transcript for the *DEFB4A* gene (ensemble.org). The illustration of the subcellular localization of the *DEFB4A* gene is shown in **Figure S1A** (Compartments.jensenlab.org/), while the illustration of its gene ontology can be shown in **Figure S1B** (Genecards.org). In addition, the *CAMP* gene is located at 3p21.31; it has four exons and a length of 1,991 nucleotides. Moreover, the *CAMP* gene has two transcripts (ensemble.org). The illustration of the subcellular localization of the *CAMP* gene is shown in **Figure S2A** (Compartments.jensenlab.org/), while the illustration of its gene ontology can be shown in **Figure S2B** (Genecards.org).

### Retrieving *DEFB4A and CAMP* gene variants

A total of 897 single-nucleotide variations could be detected in the *DEFB4A* gene (accessed 31 May 2022). Out of these SNPs, 52 were missense SNPs, 22 were synonymous SNPs, 611 were intron SNPs, 25 were in the 5′ untranslated region (UTR), and 30 were in the 3′ UTR, besides the other downstream and upstream variants. Meanwhile, for the *CAMP* gene, 831 single-nucleotide variations could be detected (accessed 27 May 2022). Out of these SNPs, 138 were missense SNPs, 78 were synonymous SNPs, 466 were intron SNPs, 7 were in the 5′ untranslated regions (UTR), and 22 were in the 3′ UTR, besides the other downstream and upstream variants.

### Investigating the effect of the SNPs on *hBD*-2 function

For *h*BD-2, among the 52 missense SNPs, 24 missense SNPs were found in amino acid sequence from position 24 to position 64 that correspond to *h*BD-2 as the first 23 amino acids represent a signal peptide (https://www.uniprot.org/uniprot/O15263). These 24 missense SNPs were investigated for their effect on protein function using six *in silico* tools (SIFT, PolyPhen-2, SNAP, PROVEAN, PHD-SNP, and SNP&GO). Only three SNPs [rs1173728551 (P44L), rs867101800 (G51D), and rs1585735110 (C53G)] were found to be deleterious by at least five tools. **Table 1** shows the detailed results for all these 24 SNPs in *h*BD-2 with the various used tools.

**Table 1:** Prediction and scores of deleterious missense SNPs by six *in silico* tools in *h*BD-2.

	SNP ID	AA change	SIFT		PolyPhen-2		PROVEAN		SNP&GO		PHD-SNP		SNAP2	
			Prediction	Score	Prediction	Score	Prediction	Score	Prediction	RI score	Prediction	RI score	Prediction	Score
1	rs1819008468	G24S	Tolerated	1	Benign	0.000	Neutral	−1.575	Neutral	6	Neutral	7	Effect	34
2	rs1819008558	G24A	Tolerated	1	Benign	0.001	Neutral	−1.879	Neutral	6	Neutral	7	Effect	36
3	rs1250045369	I25V	Tolerated	0.51	Benign	0.141	Neutral	−0.423	Neutral	7	Neutral	7	Effect	33
4	rs1187934232	D27N	Tolerated	1	Benign	0.000	Neutral	3.17	Neutral	9	Neutral	8	Effect	4
		D27H	Deleterious	0.02	Benign	0.025	Neutral	−0.689	Neutral	6	Neutral	7	Effect	66
5	rs1585735067	T30P	Deleterious	0.02	Possibly damaging	0.575	Deleterious	−3.942	Disease	2	Neutral	6	Effect	73
6	rs867028308	L32I	Tolerated	0.38	Benign	0.025	Neutral	−0.392	Neutral	8	Neutral	8	Effect	16
7	rs1207680444	L32R	Tolerated	0.34	Possibly damaging	0.790	Neutral	−1.597	Neutral	5	Disease	3	Effect	43
8	rs867852480	S34C	Tolerated	0.06	Probably damaging	0.963	Deleterious	−3.663	Neutral	4	Neutral	6	Effect	56
9	rs868395018	S34N	Tolerated	1	Possibly damaging	0.787	Neutral	2.195	Neutral	8	Neutral	5	Neutral	-11
10	rs1238855923	A36G	Tolerated	1	Probably damaging	0.918	Neutral	2.705	Neutral	9	Neutral	6	Neutral	0
11	rs771000730	P40L	Tolerated	0.19	Probably damaging	0.975	Deleterious	−6.617	Neutral	6	Disease	2	Effect	71
12	rs1819009406	P44S	Tolerated	0.26	Probably damaging	0.960	Deleterious	−5.217	Neutral	2	Neutral	4	Effect	43
13	**rs1173728551**	**P44L**	**Deleterious**	**0.05**	**Probably damaging**	**0.975**	**Deleterious**	**−6.65**	**Neutral**	**5**	**Disease**	**1**	**Effect**	**9**
14	rs1819009507	R45T	Tolerated	0.57	Benign	0.218	Neutral	−1.273	Neutral	2	Neutral	0	Effect	33
15	rs867423555	R46G	Tolerated	0.43	Benign	0.117	Neutral	0.625	Neutral	6	Neutral	0	Effect	36
16	rs1819009626	R46T	Tolerated	0.59	Benign	0.088	Neutral	−0.849	Neutral	1	Disease	1	Effect	24
17	rs1819009676	Q49H	Deleterious	0.03	Benign	0.005	Deleterious	−3.902	Neutral	7	Neutral	8	Effect	41
18	rs1819009719	I50F	Tolerated	0.06	Possibly damaging	0.494	Deleterious	−3.667	Disease	6	Neutral	4	Effect	79
**19**	**rs867101800**	**G51D**	**Deleterious**	**0**	**Probably damaging**	**0.989**	**Deleterious**	**−7**	**Disease**	**5**	**Disease**	**1**	**Effect**	**91**
**20**	**rs1585735110**	**C53G**	**Deleterious**	**0**	**Possibly damaging**	**0.849**	**Deleterious**	**−12**	**Disease**	**6**	**Disease**	**2**	**Effect**	**93**
21	rs867683988	T58A	Tolerated	0.05	Benign	0.075	Neutral	−0.592	Neutral	9	Neutral	9	Effect	59
22	rs1161262387	K59Q	Deleterious	0.05	Probably damaging	0.937	Deleterious	−3.133	Neutral	5	Neutral	6	Effect	83
23	rs866179345	K63N	Deleterious low confidence	0.04	Benign	0.003	Deleterious	−2.82	Neutral	9	Neutral	8	Effect	70
24	rs866773137	P64A	Deleterious low confidence	0	Possibly damaging	0.811	Neutral	−1.262	Neutral	10	Neutral	8	Effect	46

Bold font represents the selected most harmful SNPs.

### Investigating the effect of the SNPs on LL-37 function

For LL-37, 27 missense SNPs were found in amino acid sequence from position 134 to position 170 that correspond to the mature LL-37 (https://www.uniprot.org/uniprot/P49913). These 27 missense SNPs were investigated for their effect on protein function using six *in silico* tools (SIFT, PolyPhen-2, SNAP, PROVEAN, PHD-SNP, and SNP&GO). None of these SNP was found to be deleterious by at least five tools. **Table S1** shows the detailed results for all these 27 SNPs in LL-37 with the various used tools.

### The analysis of the variants’ effects on the stability of *h*BD-2

The analysis of the variants’ effects on the stability of *h*BD-2 was performed using the I-Mutant 2.0 tool in addition to the Mu-Pro tool. All the three SNPs were found to decrease the stability of *h*BD-2 by both I-Mutant 2.0 and Mu-Pro tools. **Table 2** shows the detailed results and the values of the analysis.

**Table 2:** Effects on *h*BD-2 stability with missense mutations.

SNP Id	AA change	I-mutant 2			MU-pro	
		I-mutant 2 prediction	Reliability Index (RI)	DDG value (kcal/mol)	Prediction	delta delta G
rs1173728551	P44L	Decrease	6	−0.99	Decrease stability	−0.12301499
rs867101800	G51D	Decrease	8	−1.24	Decrease stability	−0.80760408
rs1585735110	C53G	Decrease	6	−0.42	Decrease stability	−1.441339

### The recognition of the location of the SNPs on *h*BD-2 domains

The analysis of *h*BD-2 by InterPro showed the existence of a domain called Beta/alpha-defensin, C-terminal domain (InterPro entry: IPR006080). The investigation of the locations of our three variants was performed, and the three SNPs were shown to be located in this domain (**Table 3**).

**Table 3:** Locations of the selected residues on *h*BD-2 domains, ConSurf conservation analysis, and predicted secondary structure.

SNP Id	AA change	Location on protein	ConSurf conservation analysis	Secondary structure
rs1173728551	P44L	Beta/alpha-defensin, C-terminal domain (IPR006080)	Variable position	Coil
rs867101800	G51D		Highly conserved	Strand
rs1585735110	C53G		Highly conserved	Strand

### The investigation of the phylogenetic conservation of *h*BD-2 residues

The residues of the *h*BD-2 protein were analyzed for their phylogenetic conservation using the ConSurf tool. Two SNPs (G51D and C53G) were found to be positioned in highly conserved locations while the other SNP (P44L) was found to be positioned in a variable location (**Table 3**).

### Secondary structure analysis

**Figure 1:**
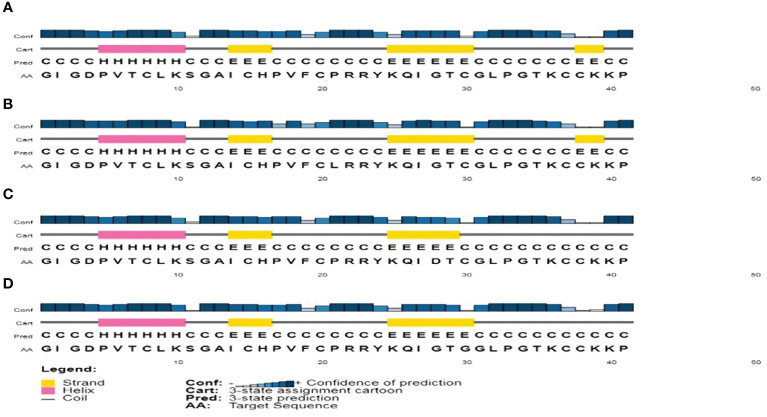
The analysis of secondary structure as produced by the PSIPRED tool. **(A)** The analysis of the secondary structure of *h*BD-2 (wild type). **(B)** The analysis of the secondary structure of *h*BD-2 with P44L SNP. **(C)** The analysis of the secondary structure of *h*BD-2 with G51D SNP. **(D)** The analysis of the secondary structure of *h*BD-2 with C53G SNP.

The PSIPRED method was used for predicting the secondary structure of *h*BD-2 as shown in **Figure 1A**. The secondary structures at residues 44, 51, and 53 were found to be coil, strand, and strand, respectively, as shown in **Table 3**. Furthermore, the secondary structures of *h*BD-2 were predicted with our mutations as shown in **Figure 1**. On one side, P44L was not found to lead to changes in the secondary structure of *h*BD-2 (**Figure 1B**). On the other side, G51D and C53G mutations were found to be associated with differences in the secondary structures of HBD-2; both mutations were associated with loss of the strand structure at the end of the protein as shown in **Figures 1C, D**, respectively.

### Analyzing the effects of our variants on *h*BD-2 protein structure

**Figure 2:**
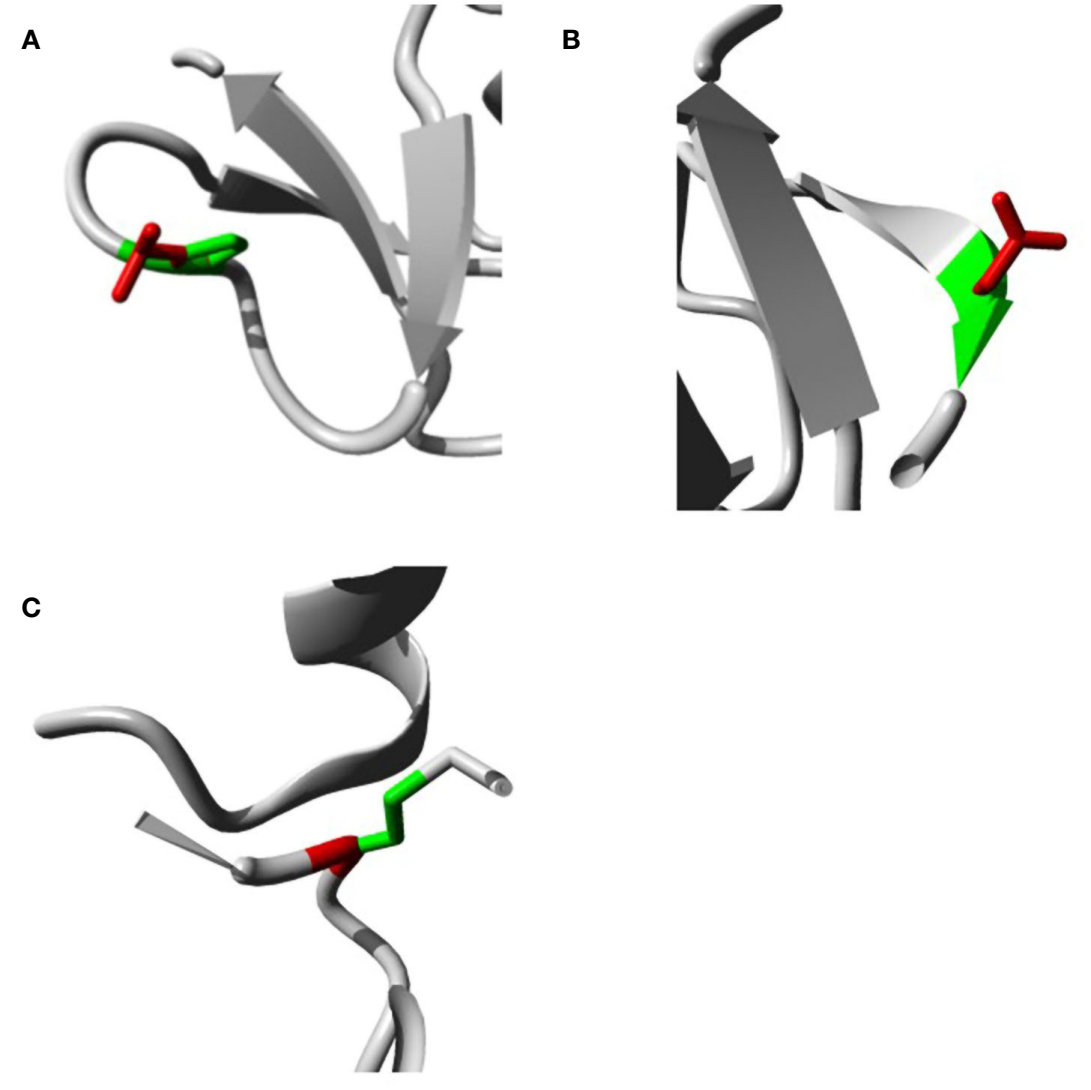
Impacts of SNPs on *h*BD2 3D structure as produced by the HOPE bioinformatics tool. **(A)** Impacts of SNPs on *h*BD-2 3D structure with P44L SNP. **(B)** Impacts of SNPs on the *h*BD-2 3D structure with G51D SNP. **(C)** Impacts of SNPs on the *h*BD-2 3D structure with C53G SNP.

The analysis was extended to study the effects of our variants on *h*BD-2 protein structure using the HOPE tool. The structural effects in *h*BD-2 protein with the substitution of the amino acids are described in detail in **Table 4**. **Figures 2A–C** illustrate the replacement of the wild-type residue with the mutant one for P44L, G51D, and C53G, respectively.

**Table 4:** The predicted SNPs’ impacts on the *h*BD-2 structure by the HOPE server.

SNP ID	AA change	Amino acid properties	Location/Structure	SNP’s impact on the protein
**rs1173728551**	**P44L**	The mutant amino acid has a bigger size than the wild-type one; being at the surface of the protein, the mutation of this residue could lead to disturbance in the interactions of the protein.	The rigidity of the wild-type proline is responsible for a specific backbone conformation; therefore, this mutation could lead to disturbance in this conformation. In addition, this mutation is very adjacent to a cysteine bond, which could be affected by this mutation.	The mutated residue is found at the surface of the main domain of this protein. However, the conservation analysis suggests the absence of a damaging effect on the protein with the mutated residue.
**rs867101800**	**G51D**	The introduced charge by the mutant amino acid could cause repulsion between this amino acid and the adjacent ones. Moreover, the mutant amino acid has a bigger size than the wild-type one; being at the surface of the protein, the mutation of this residue could lead to disturbance in the interactions of the protein. In addition, as glycine with its flexibility is important to the unusual torsion angle, this mutation could lead to inappropriate conformation and disturbance in the local structure.	The mutation will lead to the loss of the flexibility of glycine, which could be important to the function of the protein.	The mutated residue is found at the surface of the main domain of this protein. In addition, the wild-type residue is a highly conserved one, so that the mutation is expected to be a damaging one.
**rs1585735110**	**C53G**	The mutation will lead to the occurrence of empty space inside the core of this protein due to the smaller size of the mutated residue. In addition, the hydrophobic interactions at this position could be lost due to this mutation.	The mutation will lead to the loss of the cysteine bridge (formed by the wild-type cysteine) and will cause serious damage to the protein’s structure consequently. Moreover, the flexibility of the mutant-type glycine could disturb the needed rigidity.	The mutation could disturb the core structure of the domain as it is buried in the core of this domain. In addition, the wild-type residue is a highly conserved one, so that the mutation is expected to be a damaging one.

### Molecular Docking and binding pose prediction

**Figure 3:**
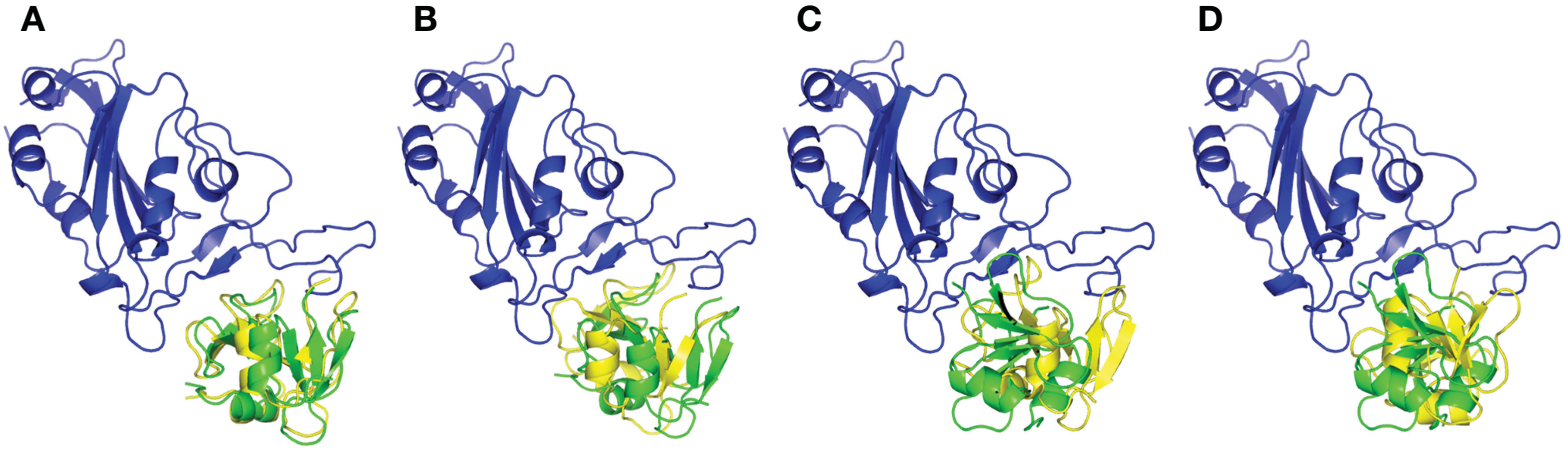
Aligned structures obtained from different docking servers for the docked *h*BD-2 dimeric forms at the SARS-CoV-2 RBD interface. Docked *h*BD-2 dimers generated from ClusPro (green cartoon) and ZDOCK (yellow cartoon) were aligned at the binding interface of the SARS-CoV-2 RBD (blue cartoon). **(A)** native *h*BD-2 model, **(B)** P44L *h*BD-2 mutant model SNP1, **(C)** G51D *h*BD-2 mutant model SNP2, and **(D)** C53G *h*BD-2 mutant model SNP3.

Both employed docking servers illustrated relevant binding of the four different *h*BD-2 dimers (one native and three SNP variants; SNP1 to SNP3) at the spike RBD interface. For simplicity, the *h*BD-2 SNP-variant dimers, P44L, G51D, and C53G, would be referred to as SNP1, SNP2, and SNP3, respectively, within the forthcoming context. Examining the top complexes obtained from each server revealed comparable protein arrangement based on the RMSD alignment analysis with values of 0.780 Å, 0.730 Å, 0.934 Å, and 0.816 Å for the native, SNP1, SNP2, and SNP3 docked complexes, respectively (**Figure 3**). Analyzing the complex interfaces *via* PDBePISA illustrated a larger interface solvent-accessible area as well as interaction contacts (# hydrogen bonds and # salt bridges) for the complexes obtained from the ZDOCK server over those from ClusPro (**Table 5**). The depicted preferentiality of the ZDOCK-generated complex was reasonably translated into higher negative Δ^1^G scores as well as lower Δ^1^G *p*-value implying profound interaction specificity for the *h*BD-2 towards the spike RBD interface surface being of higher hydrophobicity than would-be-average for given structures. Based on the above findings, ZDOCK-obtained complexes were considered more significant for further interface evaluation and to be used for subsequent molecular dynamics simulation studies.

**Table 5:** Descriptors of the spike RBD/*h*BD-2 interface analysis predicted *via* PDBePISA server.

Dock Sever	*h*BD-2 genotype	Spike RBD		*h*BD-2		Interface				
		Interface Residues	Interface Surface ^a^ (Å^2^)	Interface Residues	Interface Surface ^a^ (Å^2^)	Interface Surface ^b^ (Å^2^)	# H-bonds	# Salt Bridges	Δ^i^G ^c^ (kcal/mol)	Δ^i^G *p*-Value ^d^
ClusPro	**Native**	30	19,868	24	5,652	930	10	3	−8.6	0.701
	**SNP1**	27	20,192	20	5,818	967	8	2	−5.7	0.508
	**SNP2**	38	20,066	28	5,715	1,077	9	1	−6.3	0.628
	**SNP3**	37	19,924	22	5,579	1,060	9	0	−6.0	0.647
ZDOCK	**Native**	30	20,192	23	5,735	1,112	12	4	−10.2	0.426
	**SNP1**	35	19,938	25	5,882	1,002	9	2	−8.2	0.470
	**SNP2**	40	20,192	22	5,946	1,124	9	2	−9.6	0.571
	**SNP3**	40	20,192	27	5,951	1,092	8	2	−6.7	0.581

^a^ Solvent-accessible surface area within squared angstrom units for each bound protein.

^b^ Interface area denotes the difference in accessible surface areas of isolated and interfacing structures divided by two.

^c^ Δ^1^G denotes the gained solvation-free energies through interface formation. Higher negative values imply hydrophobic interfaces and positive protein affinity.

^c^ Δ^1^G p-value denotes the p-value of the gained solvation-free energies. It measures the probability of getting a lower Δ^1^G than the observed Δ^1^G when the interface atoms are randomly picked from the protein’s surface. Δ^1^G p-values below 0.50 imply that the interfaces of high hydrophobicity are even higher than would-be-average for given structures, which further implies that the interface surface could be interaction-specific. # means number of.

**Figure 4:**
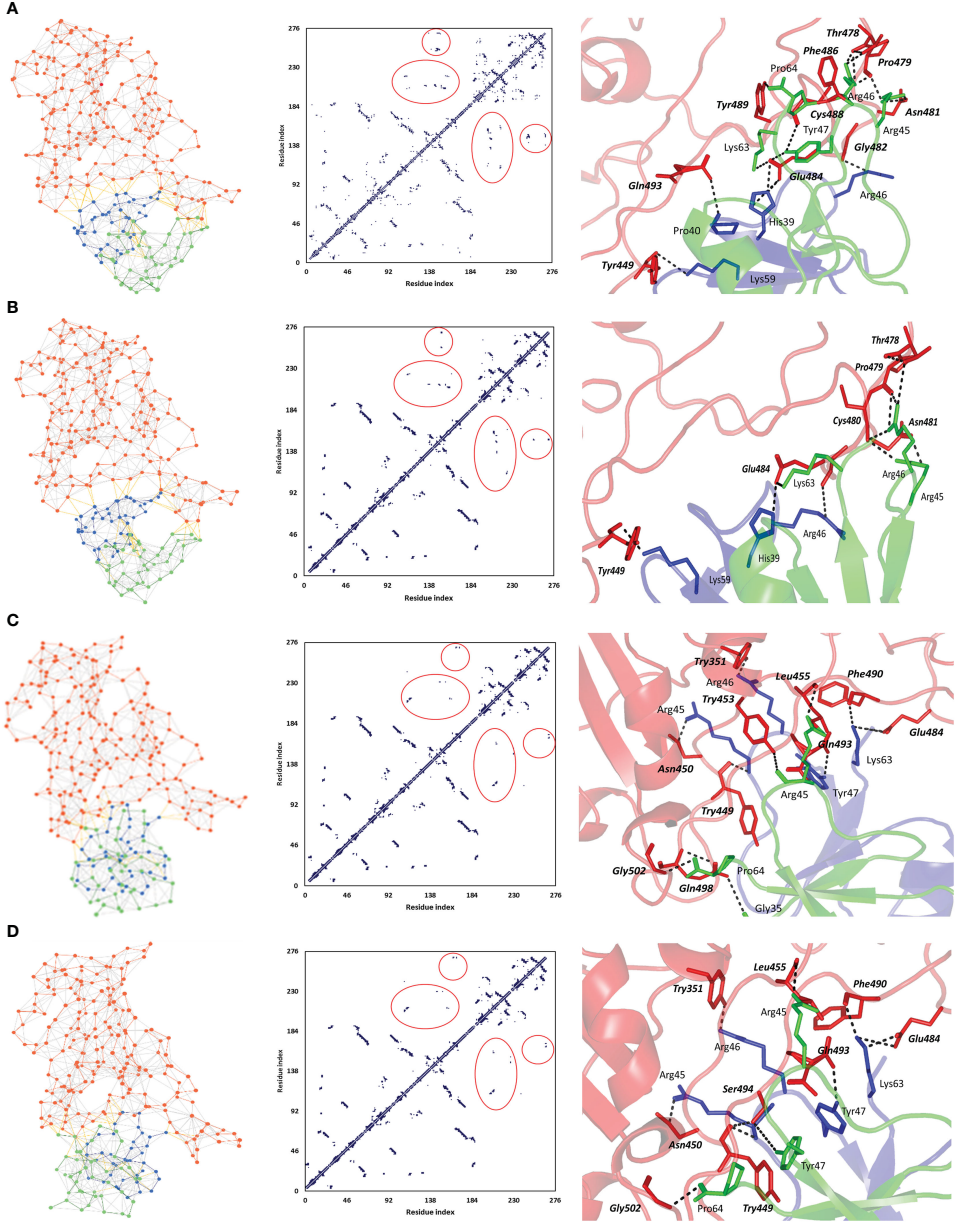
Interface analysis for the ZDOCK-obtained spike RBD/*h*BD-2 dimer complexes using NASP sever. **(A)** Native *h*BD-2 model, **(B)** SNP1 *h*BD-2 mutant model, **(C)** SNP2 *h*BD-2 mutant model, and **(D)** SNP3 *h*BD-2 mutant model. Left panels illustrate the network between edges (inter-/intra-molecular bonds as yellow and gray colors, respectively) and dots/nodes (amino acids; orange for spike RBD and blue/green for the *h*BD-2 dimers). The middle panels illustrate the contact plots for the residue–residue interactions between the monomeric units of spike RBD (residue index 1–194) and *h*BD-2 (residue index 195–276). Circle highlights are shown to pinpoint the contact interfacing residues between spike RBD and *h*BD-2 proteins, as well as *h*BD-2 protein monomers. Right panels illustrate the interface key polar interactions as predicted by the PDBePISA docking server. Protein backbones are depicted as a transparent cartoon colored red, blue, and green for spike RBD, *h*BD-2 protomer A, and *h*BD-2 protomer B, respectively. Key residue pairs are presented as lines, colored depending on their location within the proteins, and numbered according to their respective residue sequence. Spike RBD residues are presented in bold and italic. Bonds of polar interactions are black dashed lines.

A general binding affinity trend has been illustrated with the ZDOCK-generated complexes where both Δ^i^G and # polar interface interactions were more favored for the binding affinity of the native *h*BD-2 dimer as compared to the SNP variants (**Table 5**). This trend was quite consistent with complex structural network analysis findings obtained from the NAPS server since # nodes (residues) and edges (bonds) were reduced with the SNP variants as compared to the native *h*BD-2 form (**Figure 4**; left panels). Applying 7 Å as the upper threshold and one residue for separation index, the estimated node:edge ratios were 282:1,149, 277:1,030, 276:1,116, and 277:1,120 for the native *h*BD-2, SNP1, SNP2, and SNP3 bound complexes, respectively. Generally, the reduction within the edge numbers would correlate to compromised protein–protein binding interactions as well as less favored complex stability (60, 67). Generated inter-/intra-molecular contact plots showed lower interaction patterns with the *h*BD-2 SNP variants in relation to those exhibited by the native congruent form (**Figure 4**; middle panels). These latter patterns were delineated by circle highlights placed on **Figure 4** where these circle highlights show higher native spike RBD/*h*BD-2 molecular interactions as compared to those of SNP variants.

Key binding residues at the interface between the spike RBD and investigated *h*BD-2 dimeric forms are shown in **Figure 4** (right panels). Several common polar residues involved in spike RBD binding with native and SNP1 *h*BD-2 interface include spike *Thr478*, *pro479*, *Asn481*, and *Glu484*, which mediated hydrogen bond interactions with *h*BD-2 residues His39, Arg45, Arg46, and/or Tyr47 belonging to either protomer A, protomer B, or both (**Table 6**; spike residue will be written in italic formate from here forward). On the other hand, amino acids such as *Tyr351*, *Asn450*, *Tyr453*, *Phe490*, *Gln493*, and *Gly502* were significant for both SNP2 and SNP3 *h*BD-2 complex stabilities as they furnished hydrogen bonding with Arg45, Arg46, Tyr47, Lys63, and Pro64 of either one or both protomers. Interestingly, binding with spike *Tyr449* was common for all four complex interactions where it mediated strong polar hydrogen bond interactions with Lys59 of native and SNP1 *h*BD-2 proteins. On the other hand, spike *Tyr449* was significant for Arg45–hydrogen bond association at SNP2 and SNP3 proteins. The latter spike–Tyr449 direct polar interactions were directed towards the *h*BD-2 protomer A chain, rather than the protomer B one. Moreover, *h*BD-2 protomer A Arg46 and protomer B Arg45 and Pro64 amino acids were the most frequent residues involved with spike RBD interface interaction and binding. As these residues were in close proximity to SNP residues (P44L, G51D, and C53G), this could highlight the influence of these mutated residues on the conformation of their neighboring key interacting residues and, thus, in turn, the binding pose, orientation, and affinity of the simulated *h*BD-2.

**Table 6:** Key interface per-residue polar interactions *via* PDBePISA and predicted MM/GBSA binding energies.

*h*BD-2 genotype	Polar interactions		MM/GBSA calculations (kcal/mol)		
	Hydrogen bonds	Salt bridges	Total binding energy	Per-residue contributions^a^(≥1.50 kcal/mol)	
		RBD	*h*BD-2	
**Native**	*Gln493*[NE2] A:Pro40[O] 3.54Å *Glu484*[OE2] A:His39[NE2] 2.11Å *Gly482*[O] A:Arg46[NE] 2.67Å *Tyr449*[O] A:Lys59[NZ] 3.68Å *Phe486*[N] B:Arg46[O] 2.06Å *Tyr489*[OH] B:Pro64[O] 2.33Å *Asn481*[OD1] B:Arg45[NH1] 2.72Å *Asn481*[OD1] B:Arg45[NH2] 2.80Å *Thr478*[OG1] B:Arg46[NH1] 2.38Å *Pro479*[O] B:Arg46[NH2] 2.04Å *Glu484*[OE1] B:Tyr47[OH] 2.92Å *Cys488*[O] B:Tyr47[OH] 3.59Å	*Glu484*[OE2] A:His39[ND1] 3.95Å *Glu484*[OE1] A:His39[NE2] 3.01Å *Glu484*[OE2] A:His39[NE2] 2.11Å *Glu484*[OE1] B:Lys63[NZ] 3.27Å	−53.16	*Glu484* *Tyr489* *Phe490* *Phe486* *Tyr449* *Ans481* *Val483* *Glu471*	A:Phe42 A:Pro56 A:Arg45 A:Val41 A:His39 B:Tyr47 B:Pro64 A:Pro44 B:Lys63 B:Arg45
**SNP1**	*Glu484*[OE2] A:His39[NE2] 2.74Å *Gly482*[O] A:Arg46[NE] 3.68Å *Tyr449*[O] A:Lys59[NZ] 3.21Å *Asn481*[O] B:Arg45[NH2] 3.48Å *Cys480*[O] B:Arg45[NH2] 3.07Å *Thr478*[OG1] B:Arg46[NH1] 3.59Å *Pro479*[O] B:Arg46[NH1] 2.19Å *Cys480*[O] B:Arg46[NH2] 2.60Å *Asn481*[OD1] B:Arg46[NH2] 3.71Å	*Glu484*[OE2] A:His39[NE2] 2.74Å *Glu484*[OE1] B:Lys63[NZ] 3.99Å	−40.18	*Tyr449* *Phe490* *Pro479* *Val483* *Glu484* *Glu471* *Phe486* *Thr470* *Ile472*	A:Phe42 A:Pro56 A:Arg45 A:Val41 A:Leu44 B:Lys63 B:Arg46B:Tyr47A:His39
**SNP2**	*Gln493*[NE2] A:Tyr47[OH] 2.85Å *Tyr449*[O] A:Arg45[N] 2.46Å *Asn450*[O] A:Arg45[NH1] 3.06Å *Tyr351*[OH] A:Arg46[NH2] 2.13Å *Phe490*[O] A:Lys63[NZ] 2.91Å *Gln498*[NE2] B:Gly35[O] 3.83Å *Tyr453*[OH] B:Arg45[O] 3.64Å *Gly502*[N] B:Pro64[OXT] 2.34Å *Leu455*[O] B:Arg45[NH2] 2.93Å	*Glu484*[OE2] A:Lys63[NZ] 3.55Å *Glu484*[OE1] A:Lys63[NZ] 3.10Å	−14.23	*Tyr449* *Tyr505* *Asn501* *Gly502* *Phe490* *Asn450* *Leu452* *Leu455* *Phe497*	A:Phe42 B:Tyr47 A:Arg45 A:His39 A:Pro44A:Val41
**SNP3**	*Gln493*[NE2] A:Tyr47[OH] 2.81Å *Tyr449*[O] A:Arg45[N] 2.45Å *Asn450*[O] A:Arg45[NH1] 3.06Å *Tyr351*[OH] A:Arg46[NH2] 2.11Å *Phe490*[O] A:Lys63[NZ] 2.89Å *Gly502*[N] B:Pro64[OXT] 2.35Å *Leu455*[O] B:Arg45[NH2] 2.90Å *Ser494*[O] B:Tyr47[OH] 3.87Å	*Glu484*[OE2] A:Lys63[NZ] 3.55Å *Glu484*[OE1] A:Lys63[NZ] 3.10Å	−17.54	*Tyr449* *Tyr505* *Asn501* *Gly502* *Asn450* *Leu452* *Phe490* *Leu455*	A:Phe42 B:Tyr47A:Arg45 A:Pro44 A:His39

A and B denote hBD-2 protomer A and B, respectively.

^a^Per-residue MM/GBSA energy contributions that are listed in descending order.

On similar bases, multiple salt bridges between spike *Glu484* at one side and His39 and Lys63 of either *h*BD-2 protomer were almost consistent across the four ZDOCK-generated docked complexes. Besides polar interacting residues, hydrophobic interface residues at spike RBD showed significant closeness and relevant non-polar contacts with neighboring *h*BD-2 amino acids. Spike residues such as *Val483*, *Phe490*, *Leu452*, and *Ile472* depicted ≤5.0 Å distances from the respective Tyr47, Phe42, and Val41 amino acids at native and SNP1 *h*BD-2 protomers. Notably, spike *Val583* showed hydrophobic contact with native Pro44 and its SNP1 variant residue, Leu44, with a much closer distance toward the earlier native residue (3.9 Å versus 4.5 Å αC distance). Regarding both SNP2 and SNP3 variants, residues such as Pro64, Phe42, His39, Ile37, Tyr47, and Val41 illustrated potential hydrophobic interaction with *Ile472*, *Val483*, *Tyr489*, *Tyr449*, *Tyr505*, and/or *Val445* of the spike protein. For translating all the above preferential per-residue interactions into respective binding energy terms, the MM/GBSA binding energy calculations were applied for the top four spike RBD/*h*BD-2 complexes. Higher binding energy was assigned for the native *h*BD-2 complex as compared to its SNP variants. As expected, higher residue-wise energy contributions were assigned for the key interacting residues of both spike RBD and *h*BD-2 proteins (**Table 6**). Focusing on the mutant *h*BD-2 SNP residues, the residues were of lower negative energy contributions than their native amino acids (Pro44 −2.39 kcal/mol vs. Leu44 −1.35 kcal/mol in SNP1; Gly51 −0.13 kcal/mol vs. Asp51 0.21 kcal/mol in SNP2; Cys53 −0.14 kcal/mol vs. Gly53 0.02 kcal/mol in SNP3). Interestingly, mutant residues at SNP2 and SNP3 depicted positive repulsive energy contributions rather than favored attractive ones.

### All-atom molecular dynamics simulation and thermodynamic stability

**Figure 5:**
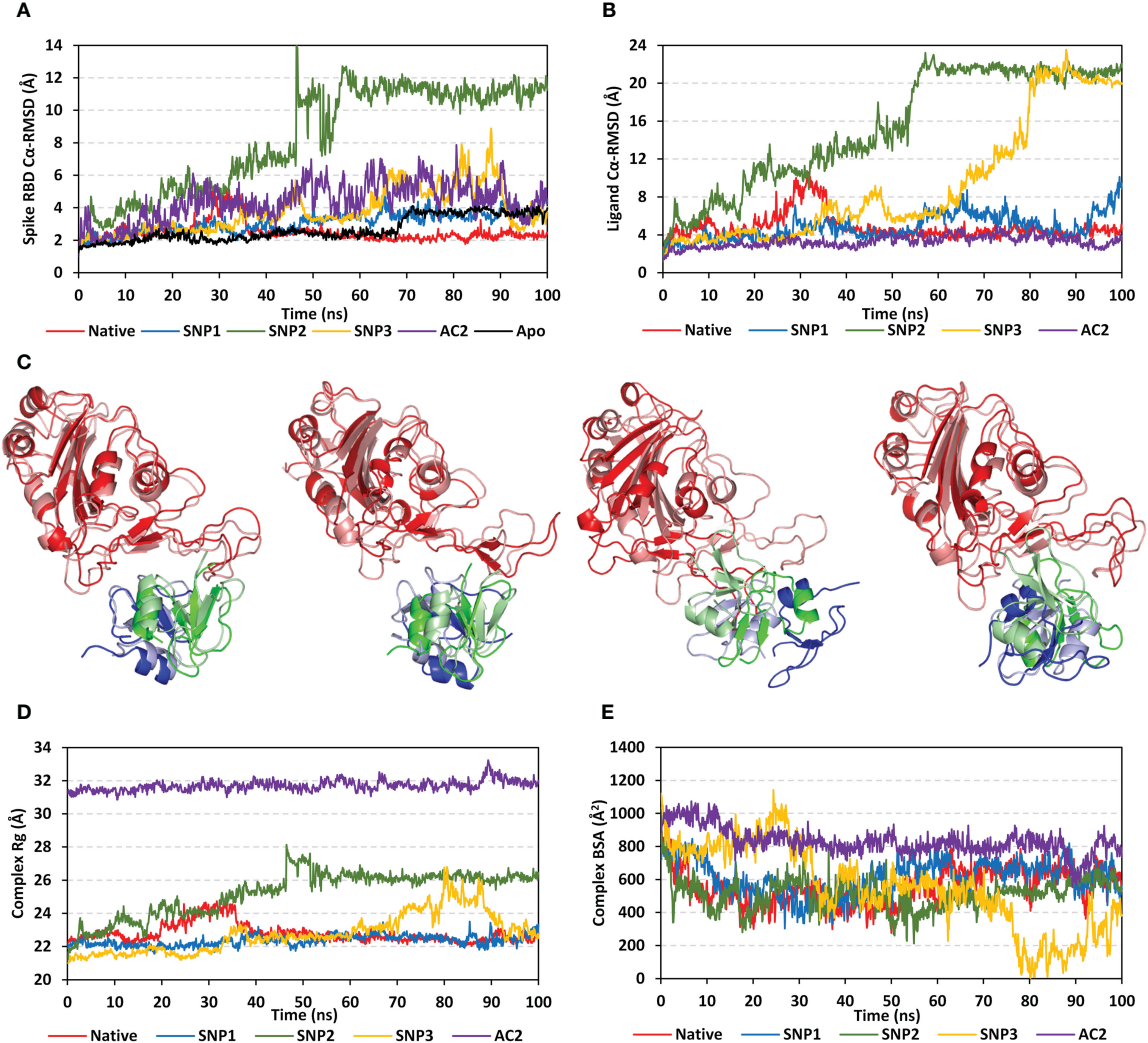
Stability analysis of the simulated spike RBD/ligand complexes across the molecular dynamics simulations. **(A)** Spike RBD Cα-atom RMSDs; **(B)** ligand Cα-atom RMSDs, as a function of the simulation times (ns). **(C)** Overlaid spike RBD/*h*BD-2 complex snapshots at 0 and 100 ns for native, SNP1, SNP2, and SNP3 ligand proteins from the left to the right side, respectively. Proteins are represented as cartoons and colored red, blue, and green for respective spike RBD, *h*BD-2 protomer A, and *h*BD-2 protomer B domains. Initial and final extracted frames were represented in faint or dark colors, respectively. **(D)** Complex Rgs; **(E)** Complex BSAs, as a function of the simulated times (ns).

The RMSD trajectories of both spike RBD and *h*BD-2 proteins were monitored across the 100-ns all-atom simulation runs in reference to the alpha-carbon atoms (αC) of their respective initial structure. The spike RBD αC-RMSD showed deferential tones based on the ligand-bound protein (**Figure 5A**). The steadiest RMSD tones were depicted for the RBD in complex with the native *h*BD-2 ligand as compared to any other simulated RBD proteins. Despite limited fluctuations around 25- to 35-ns timeframes, native-bound RBD was maintained around an average RMSD value (2.49 ± 0.59 Å) for more than half the simulation time. On the other hand, RBD bound with *h*BD-2 SNP variants were at higher RMSD values (SNP1 3.09 ± 0.65 Å; SNP2 8.27 ± 3.23 Å; SNP3 3.75 ± 1.32 Å) with shown fluctuations across the simulation times. Across different *h*BD-2/RBD models, the highest fluctuations were assigned to the SNP2-bound RBD reaching up to 11.25 Å at the end of the simulation run.

Interestingly, the RMSD trajectories of spike RBD in complex with the crystallized *h*ACE2 protein showed higher and more fluctuating tones (4.34 ± 1.17 Å) as compared to the native *h*BD-2 model. Moreover, the unliganded/apo spike RBD protein was of steady RMSD trajectories till 70 ns (2.16 ± 0.30 Å), after which they significantly increased and leveled off around 3.74 Å till the end of the simulation run reaching higher values than the native *h*BD-2/bound RBD. Monitoring the ligand RMSDs of either the bound *h*BD-2 or *h*ACE2 proteins has shown comparable patterns in regard to their respective bound spike RBD proteins (**Figure 5B**). Both the native *h*BD-2 dimer and *h*ACE2 protein units depicted the steadiest tones for more than 50-ns run times (4.24 ± 0.39 Å and 3.59 ± 0.51 Å, respectively). Higher fluctuating RMSD tones were assigned for SNP *h*BD-2 dimers showing SNP2 with the highest fluctuating values, followed by SNP3 and SNP1 proteins (15.39 ± 6.04 Å, 9.26 ± 6.39 Å, and 4.82 ± 1.35 Å, respectively). Notably, the αC-RMSD tones of bound ligand proteins (*h*BD-2) were almost 1.5-fold higher than those of their bound spike RBD proteins, except for *h*ACE2 where the latter showed steadier RMSD tones than its bound spike RBD protein.

Conformational analysis through aligning the starting and final spike RBD/*h*BD-2 complex structures illustrated differential orientations for the bound ligand at the spike RBD interface (**Figure 5C**). Visually observed as well as correlated to the obtained RMSD trajectories, limited conformational and ordination changes were observed for the spike RBD/native *h*BD-2 complex in regard to its SNP variant models. A slight conformational shift was depicted for the native *h*BD-2 location and secondary structure showing minimal rotations of its flexible loops (aligned RMSD 1.44 Å). The SNP1 variant showed significant conformational rotation at almost 90° in regard to its initial structure, yet the whole protein was maintained at its same starting location (aligned RMSD 1.93 Å). The SNP1 conformational changes were associated with significant conformational alteration of the spike interface loop (residue range *Ala475*–*Cys488*) adopting a more elongated hairpin-like conformation. On the contrary, a more dramatic conformational and orientational shift was assigned to the SNP2 protein showing a significant drift of ~20 Å far from the spike RBD interface and towards the solvent side (aligned RMSD 8.85 Å). Such dramatic alterations caused the spike RBD interface loop (residues *Ser469*–*Pro491*) to adopt a different orientation being directed towards the solvent side in a way that appeared to track the drifted SNP2 protein. Regarding the last *h*BD-2 variant, SNP3 ligand protein was persistent at the spike interface, yet with inverted orientation (180° rotation) regarding its whole structure. Being dimeric, a moderate aligned RMSD value of 1.65 Å was assigned for the SNP3-bound system. Notably, these conformational changes were with minimal influence on the spike RBD conformation where the interface loop showed limited rotations at the end of the simulation being of a similar fashion to that observed for RBD in complex with native *h*BD-2 protein.

Tracking the above conformational changes was pursued through estimating the Rgs of spike RBD/*h*BD-2 complexes in relation to their gathered central masses. Following half of the simulation runs, both native and SNP1-bound complexes were of the lowest steady Rg tones (22.79 ± 0.57 Å and 22.36 ± 0.32 Å) till the end of the timeframes (**Figure 5D**). This was not consistent with the other simulated *h*BD-2 SNP variants, where SNP2 depicted a continuous uprising of Rg values from 20 to 50 ns before it leveled off at approximately 26.00 Å till reaching 100 ns. The SNP3-bound complex depicted significant Rg fluctuations up to 26.00 Å across the 70- to 90-ns timeframes. Concerning Rgs of the spike RBD/*h*ACE2 complex, steady but higher tones (31.70 ± 0.31 Å) were depicted across the whole simulation run the thing that would be consistent with the *h*ACE2 large mass (~110 kDa). Further conformational analysis was done by monitoring the amount of solvent-accessible surface areas being buried at the interface between both bound proteins across the simulation times.

Buried surface area (BSA; Å^2^) for each simulated complex was estimated using the following equation adopting the individual SASA of each bound protein (SASA_RBD_ and SASA*_h_*_BD-2_) as well as that of the whole bound complex; BSA = 0.5*(SASA_RBD_ + ASA*_h_*_BD-2_ – SASA_complex_) (5). Simulated spike RBD/*h*ACE2 complex showed the highest BSAs fluctuating around an average value of 831.55 ± 81.85 Å^2^, which was consistent with its larger size as compared to the investigated *h*BD-2 proteins (**Figure 5E**). Steady comparable BSA values were depicted for the simulated native and SNP1 *h*BD-2 complex systems (~600 Å^2^). Notably, both systems illustrated increased BSA beyond the 50-ns time window where they reached few hundreds just below the BSA of the RBD/*h*ACE2 complex. On the other hand, the SNP2 variant complex was seen with greater fluctuations at initial frames and ended with a low BSA average (502.61 ± 82.27 Å^2^) across the last 40-ns timeframes, indicating fewer areas being covered. Much higher BSA fluctuations were depicted with the SNP3 system reaching down to the lowest BSA values (~8.00 Å^2^) around the 80 ns before the BSA values were raised to 738.15 Å^2^ at the end of the simulation. Findings from RMSDs, Rgs, and BSAs conferred that SNP3 left the spike RBD interface across the 80 to 90 ns and finally returned to its initial location, where it adopted an inverted orientation.

**Figure 6:**
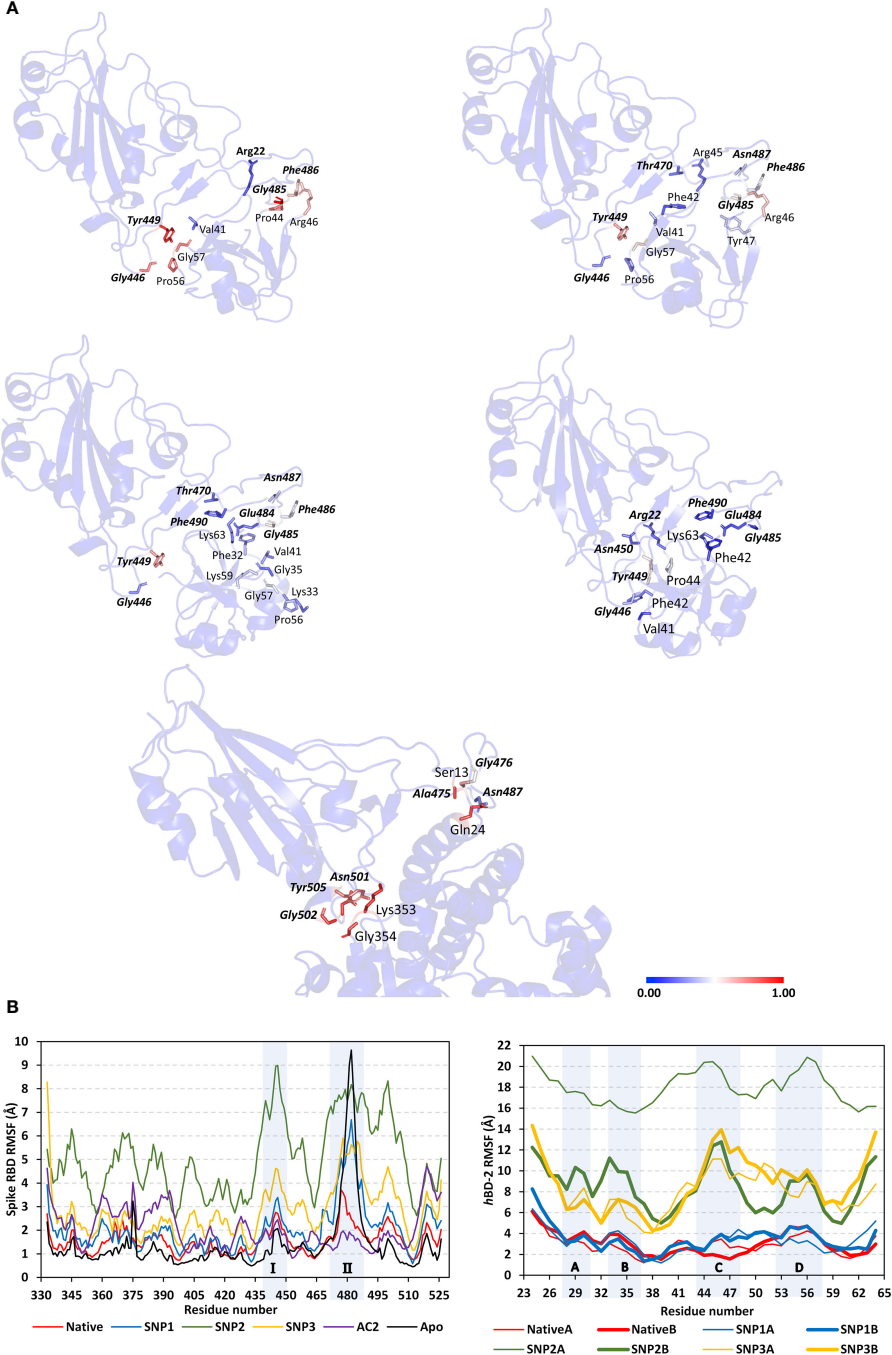
Dynamics of the simulated spike RBD/ligand complexes across the molecular dynamics simulations. **(A)** Per-residue contact distance occupancy analysis. Complexes of native *h*BD-2, SNP1, SNP2, SNP3, and *h*ACE2 (upper left, upper right, middle left, middle right, and bottom panels, respectively) are represented as cartoons highlighting residues (sticks) with relevant occupancies exhibiting contact distance at ≤5.50 Å from bound protein. Data are mapped to the residue sticks with a spectrum color bar indicating the range of 0.00 to 1.00, transitioning through 0.50 occupancies (blue, white, and red, respectively). Highlighted residues are numbered according to their respective residue sequence, while spike RBD residues are presented in bold and italic. **(B)** Spike RBD and *h*BD-2 RMSF of individual protein residues in respect to their *C*-alpha, as a function of the simulated times (ns). Shaded regions of spike RBD and *h*BD-2 residues are common regions for binding the two proteins and are thoroughly described in the above context.

For highlighting the differential contact interfaces for the simulated *h*BD-2 proteins towards their bound spike RBD target, the per-residue occupancy for each protein across the run frames was estimated, where residues achieving contact distances of ≤5.5 Å were highlighted and visually represented (**Figure 6A**). Interestingly, higher contact distance occupancies were assigned for the native *h*BD-2 dimer depicting more red-colored residues as compared to the SNP proteins. Highlighted residues (Val41, Pro44, Arg45, Arg46, Pro56, and Gly57) from native dimeric units depicted high contact distance occupancies towards two main spike RBD interface regions (I: *Leu441–Asn450* and II: *Gly476–Phe486*). The SNP1 variant residues (Val41, Phe42, Arg45, Tyr47, and Pro56) depicted relevant contact distance occupancies with both spike RBD interface residues, yet with lower occupancy values. On other hand, residues of SNP2 protein (Lys33, Val41, Phe42, Pro56, Gly57, Lys59, and Lys63), mostly at its protomer B, depicted preferential contact distance occupancies with the spike RBD interface region II. Such SNP2 preferential contact pattern is consistent with the dramatic conformational/orientation shift across the simulation run being illustrated *via* the above dynamic analysis parameters. Despite depicting relevant contacts with the two main spike RBD interface regions, residues of the SNP3 protein (Val41, Phe42, Pro44, and Arg45) as well as contacting spike ones illustrated fair contact distance occupancies, which emphasized the ligand drift from the RBD interface throughout the simulation run.

Spike interface residue *Tyr449* was shown to be significant for *h*BD-2 ligand binding as it depicted one of the highest occupancies, being the highest for native *h*BD-2, throughout each respective simulated complex. Notably, all simulated *h*BD-2 systems depicted negligible contact distance occupancies towards the anti-parcel *β*-sheets and the connecting loops (*Tyr451–Lys458* and *Pro491–Gly496*) at the spike RBD interface region being present midway between the spike’s two high occupancy regions I and II. Concerning the contact distance occupancies for the spike RBD/*h*ACE2 complex, preferential binding towards the RBD regional interface residues was also depicted with negligible values for the midway anti-parcel *β*-sheets. Nevertheless, some subtle contacting residue shifts were shown with *h*ACE2 where the RBD residues *Ala475–Thr478* and *Asn501–Tyr505*, rather than *Leu441–Asn450* and *Gly476–Phe486*, respectively, were associated with *h*ACE2 and not with *h*BD-2 dimers. Higher occupancies were for the *h*ACE2-associated regions *Asn501–Tyr505* of the bound spike RBD, while *Leu441–Asn450* residues are more significant for *h*BD-2 binding.

Thermodynamic behaviors of different *h*BD-2 and *h*ACE2 in relation to spike RBD protein were further evaluated down to their constituting residue levels *via* monitoring each respective RMSF fluctuation tones. As expected, the above-described contact residue ranges were of lower RMSFluctuations only for native and *h*ACE2-bound RBD proteins rather than those in complex with the *h*BD-2 SNP mutants (**Figure 6B**). The highest RBD RMSF tones were assigned for binding with SNP2, followed by SNP3 and SNP1, while those inbound to native *h*BD-2 and *h*ACE2 were of almost comparable immobility tones. On the other hand, differential RMSF tones were depicted for the contact interface residue ranges between the most stable holo RBD (i.e., in complex with native *h*BD-2 or *h*ACE2) and those being unliganded at their apo state. The high-occupancy contact residue range *Gly476–Phe486* was of higher flexibility (up to 9.50 Å) at its apo state as compared to those inbound with native *h*BD-2 or *h*ACE2 (~3.00 Å). On the contrary, the contact residue range *Leu441–Asn450* and *Asn501–Tyr505* were of more immobility patterns at the apo state as compared to the holo ones (~1.30 Å versus ~2.10 Å, respectively). Nevertheless, the holo/apo RMSF differences within the two latter spike RBD residue ranges were of a lower extent (0.5-fold) as compared to those obtained at the *Gly476–Phe486* residue range (>2-fold). Interestingly, only the spike RBD *Asn501*–*Tyr505* residue range was with less flexibility RMSF tones on *h*ACE2 bounding than either the native *h*BD-2-bound or even apo spike proteins. The latter highlights the significance of the spike RBD *Ala475–Thr478* residue for *h*ACE2 rather than *h*BD-2 binding. Moving towards the RMSF of the simulated *h*BD-2 proteins, higher values were assigned for the simulated SNP2 and SNP3 *h*BD-2 mutant proteins (**Figure 6B**).

Comparable RMSF trajectories were depicted for each protomer of the *h*BD-2 dimer structure, except for the SNP2 variant where its protomer A was assigned with the highest observed RMSF values (17.95 ± 1.59 Å) among all *h*BD-2 monomeric units and even those of bound spike RBD proteins. Notably, four *h*BD-2 core residue regions (A: Asp27–Cys31, B: Lys33–Ile37, C: Cys43–Lys48, D: Gly54–Thr58) showed higher RMSF tones as compared to the remaining core residues of each respective protein. The first two regions (A and B) correspond to the protein’s *N*-terminal α-helix and *β*-loop secondary structures where their depicted RMSFs were much higher in SNP2 and SNP3 variants as compared to both SNP1 and native forms. Both site A and site B were observed far from contact with the spike RBD site throughout the simulation time conferring their negligible role in *h*BD-2/RBD stability and in turn their respective high RMSFs. On the other hand, the last two *h*BD-2 regions (C and D) showed significant contact with the spike RBD site; however, high contact distance occupancies were only assigned for the native and SNP1 simulations (see **Figure 6A**). The latter could reasonably explain why sites C and D were of lower RMSFs at native and SNP1 proteins as compared to the other variants.

**Figure 7:**
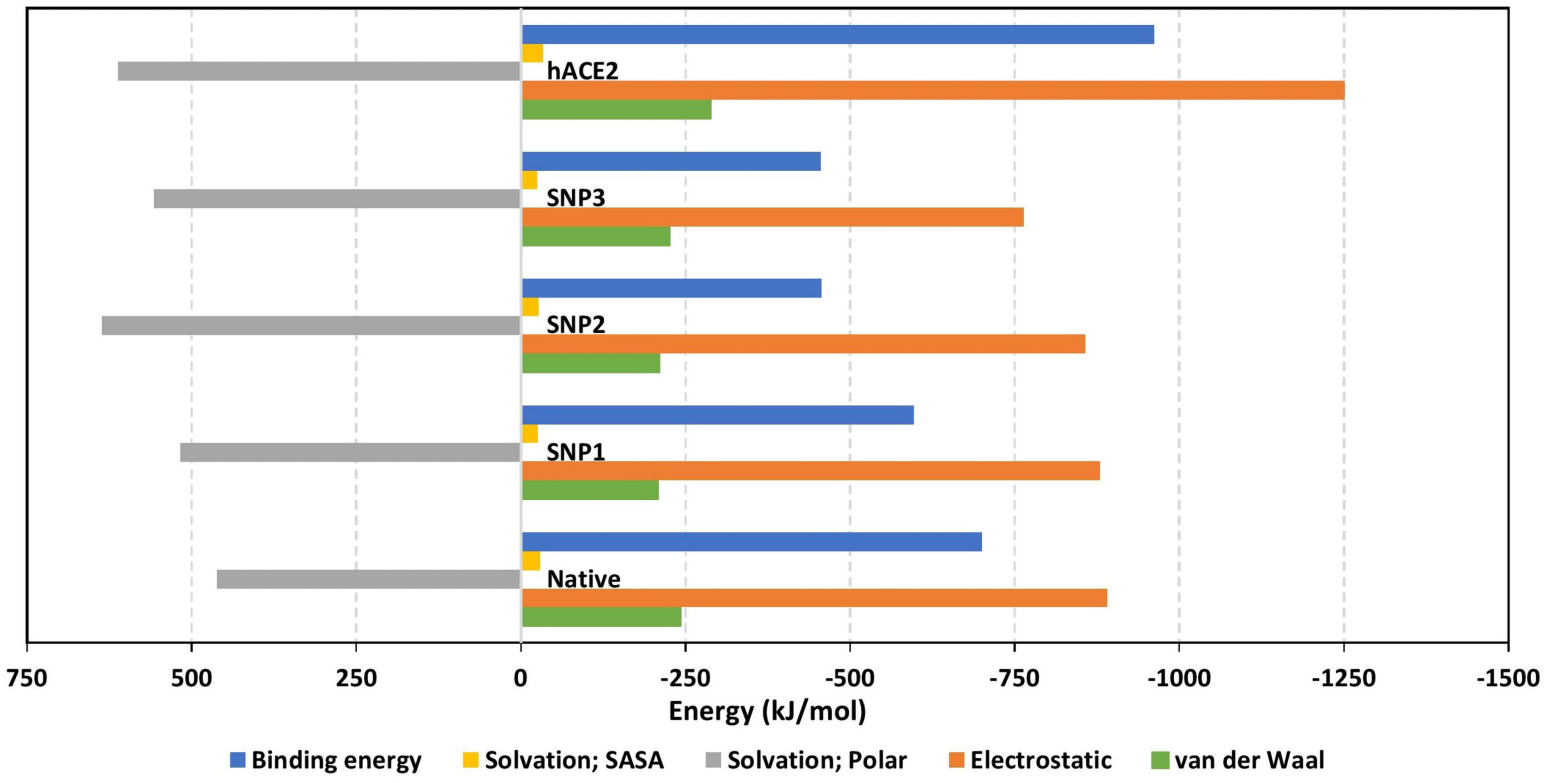
Free binding and individual energy terms for the simulated spike RBD/ligand complexes. Values are estimated in terms of kJ/mol ± SE; error bars are hidden for clarity.

**Figure 8:**
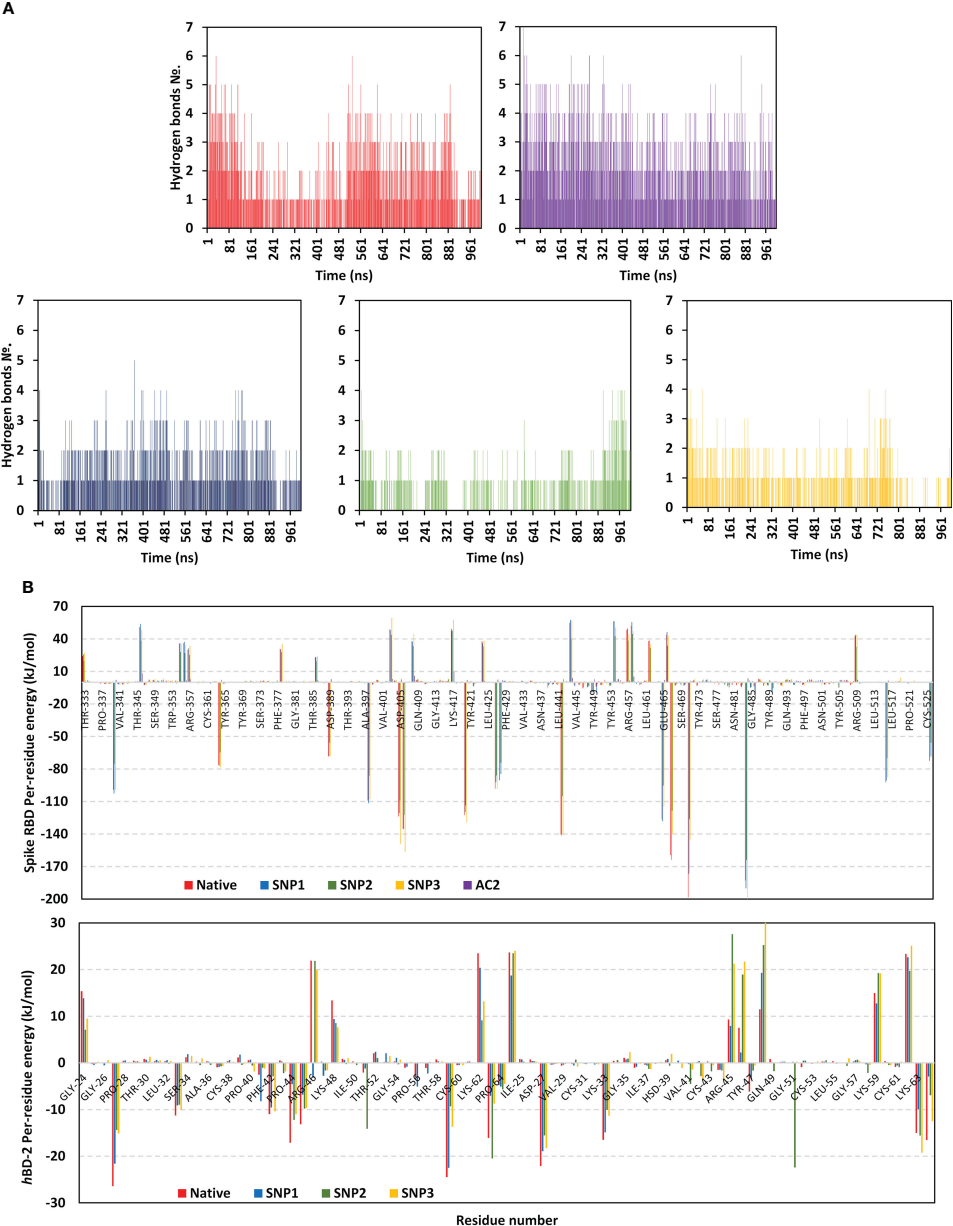
Binding interaction analysis for the simulated spike RBD/ligand complexes across the molecular dynamics simulations. **(A)** Number of furnished hydrogen bonds by the simulated complexes of native *h*BD-2, *h*ACE2, SNP1, SNP2, and SNP3 ligands (upper left, upper right, bottom left-to-right panels, respectively), as a function of the simulated times (ns). **(B)** Per-residue MM-PBSA free binding energy contributions for spike RBD and ligand proteins.

The MM/PBSA calculations revealed negative average free binding interaction energies for the native hBD-2/spike RBD complex being only second to the *h*ACE2 simulated system (**Figure 7**). The average free binding energies of SNP mutant proteins were almost half that of the spike RBD/*h*ACE2 system. Dissecting the total free energy into its constituting energy terms revealed a dominant contribution for Coulomb’s electrostatic potentials over those of the hydrophobic ones. Notably, the polar solvation penalty was significantly higher in mutant SNPs as compared to the native protein, the thing that could have compromised the mutant complex stabilities since binding is considered a solvent-displacement process. Among the simulated *h*BD-2 proteins, the native form was depicted with the highest electrostatic energy contributions, which were further illustrated by monitoring the number of formed hydrogen bonds between the proteins across the simulation runs (**Figure 8A**).

The average number of protein–protein intermolecular hydrogen bonds was higher at native *h*BD-2 (1.52 ± 1.22) in relation to its mutant variants (SNP1; 1.18 ± 0.93, SNP2; 0.62 ± 0.82, SNP3; 0.70 ± 0.82), but the earlier was only second to the *h*ACE2-associated complex (2.19 ± 1.29). Within the native *h*BD-2:RBD complex, high hydrogen bond occupancies were assigned for Lys63-Side : *Glu484-Side* (38%), Arg45-Side : *Glu484-Side* (34%), and Arg46-Side : *Glu484-Main* (22%). For SNP1, hydrogen bond pairs such as Arg46-Side : *Glu484-Main* (18%), Arg45-Side : *Glu484-Side* (17%), Arg46-Main : *Asn487-Main* (13%), and Lys48-Main : *Asn487-Side* (20%) were of lower occupancies as compared to the native protein. For the less stable SNP mutant complexes, only Arg45-Side : *Glu484-Side* (11%) and Lys63-Side : *Glu484-Side* (31%) hydrogen bond pairs at SNP2 and SNP3 complexes, respectively, were assigned with occupancies just ≥10%.

Notably, the above-described native and SNP1 hydrogen bond pairs were of reduced persistency at SNP2 and SNP3 complexes; the thing that was proposed correlated to the dramatic conformational dynamic behaviors of these latter variants. Regarding the reference complex, preferentiality of *h*ACE2 towards the spike *Ala475–Thr478* and *Asn501–Tyr505* residue ranges were further highlighted since several high-frequency hydrogen bond pairings were depicted across these regions and vicinal residues: *Tyr505-Side :* Glu37-Side (48%), *Gly502-Main :* Lys353-Main (60%), Ser19-Side : *Ala475-Main* (29%), *Lys417-Side :* Asp30-Side (20%), Tyr83-Side : *Asn487-Side* (35%), *Tyr453-Side :* His34-Side (19%), and Lys31-Side : *Gln493-Side* (13%).

Exploring the residue-wise free binding energy contribution for the simulated complex was highlighted in **Figure 8B**. Higher negative-value energy contribution was assigned for native *h*BD-2 over its mutant proteins. Preferentiality for hBD-2 to bind to spike regional I and II residues was illustrated since several constituting and vicinal residues depicted relevant negative-value energy contributions: *Asp442* (−104.91 to −141.30 kJ/mol), *Glu465* (−95.13 to −128.11 kJ/mol), *Asp467* (−118.48 to −163.76 kJ/mol), *Glu471* (−126.07 to −198.34 kJ/mol), and *Glu484* (−163.67 to −200.00 kJ/mol). In contrast, residues of the spike’s anti-parcel *β*-sheets and midway loops (*Tyr451–Lys458* and *Pro491–Gly496*) were assigned with positive repulsive energy contributions; *Arg454* (42.61 to 56.26 kJ/mol), *Arg457* (33.41 to 49.60 kJ/mol), and *Lys458* (44.60 to 55.64 kJ/mol) highlighted their unfavored binding with *h*BD-2 ligands. Per-residue energy contribution at *h*BD-2 proteins was significant for Lys33 (−8.96 to −11.25 kJ/mol), Phe42 (−2.88 to −10.97 kJ/mol), Pro44 (−4.57 to −17.11 kJ/mol), Arg45 (−6.78 to −13.16 kJ/mol), Lys59 (−9.29 to −24.48 kJ/mol), and Lys63 (−7.05 to −16.08 kJ/mol).

Focusing on comparative mutant residues with their native form, replacing Pro44 at native *h*BD-2 with Leu44 in SNP1 lowered the residue-wise energy contribution from −17.11 to −4.57 kJ/mol as well as the vicinal residue Arg45 from −13.16 to −6.78 kJ/mol. Regarding SNP2, replacing native hydrophobic Gly51 with polar Asp51 increased energy contribution (−2.03 to −14.10 kJ/mol), contributing to the dominant electrostatic potential of the ligand binding. Nevertheless, Asp28 possesses increased residue-wise size that might have contributed to steric clashes with spike residue for unfavored binding and SNP2’s depicted conformational shift. Finally, C53G SNP3 has associated more positive energy contribution in regard to its native form (1.47 versus 0.19 kJ/mol, respectively). All the above electrostatic preferentiality and comparative hydrogen bond occupancies were consistent with the obtained contact distance occupancy analysis as well as the preliminary docking results where polar residue pairs were much assigned to the native *h*BD-2 complex.

### The analysis of gene–gene interactions

The analysis of the gene–gene interaction of the *DEFB4A* gene was performed using the GeneMania tool, leading to the identification of the 20 genes with the closest connection to the *DEFB4A* gene (**Figure S3**). Among these genes, the defensin beta 103A gene (*DEFB103A)* occupied the first rank. After that, the C–C motif chemokine receptor 6 gene (*CCR6)* occupied the second rank.

## Discussion

In addition to the well-known role of *h*BD-2 and LL-37 in combatting different invading microbes and viruses (68, 69), recent studies have found an important role for *h*BD-2 and LL-37 in defending the human body against COVID-19 infection (70). Biophysical experiments and biochemical studies performed by Zhang and colleagues confirmed the ability of hBD-2 to bind the RBD of SARS-CoV-2 and inhibit the binding of this RBD to *h*ACE2 (5). These results confirmed the *in silico* findings regarding the ability of hBD-2 to bind to the RBD of SARS-CoV-2 (5). Similar results were found with LL-37 and confirmed the high affinity of its binding to the RBD of this serious virus (9, 10). The damaging missense SNPs could have serious effects on protein structure and function (71), raising questions about the fate of this preventive role against COVID infection in these cases. Therefore, we aimed to find the damaging missense SNPs for *h*BD-2 and LL-37 and study the impact of these damaging mutations on the susceptibility to this serious disease.

Beginning from 897 SNPs in the *DEFB4A* gene, 24 missense SNPs were found in the sequence corresponding to *h*BD-2. These mutations were applied to analysis with six bioinformatics tools with various approaches and algorithms to ensure the robustness of the analysis. As a result, three SNPs were predicted to be disease-causing and deleterious. On the other side, beginning with 831 SNPs in the *CAMP* gene, 27 missense SNPs were found in the sequence corresponding to LL-37. However, by applying the same six bioinformatics tools, none of these SNP was found to be deleterious by at least five tools. Consequently, none of the LL-37 SNPs was selected for further analysis and only the damaging mutations with the *DEFB4A* gene were selected for further investigation.

Due to the importance of protein stability for maintaining the functions and the structure of the protein (72), the consequences of the selected mutations on the stability of *h*BD-2 were analyzed by I-mutant 2 and MUpro tools. All three mutations were predicted to decrease the stability of the protein by both tools. Moreover, the functional analysis of the protein was performed to determine its important domains depending on the InterPro tool. The analysis revealed the presence of a domain called (Beta/alpha-defensin, C-terminal domain), on which all the three mutations were located. After that, the phylogenetic conservation was analyzed using the ConSurf server showing that G51D and C53G SNPs were positioned on highly conserved residues while P44L SNP was positioned on variable residue. As the functionally and structurally important amino acids usually display high phylogenetic conservancy (38), the occurrence of SNPs in these conserved residues is expected to have an impact on the structure or function of the protein. In addition, using PSIPRED to analyze the secondary structure revealed the presence of changes in the secondary structures of *h*BD-2 with G51D and C53G mutations. The importance of the secondary structure of a protein is manifested in its essential roles in the structure and the folding of the protein (73). The effects of the three SNPs on *h*BD-2 structure were analyzed by the HOPE server showing the damaging effects of the three mutations on *h*BD-2 structure, in particular with G51D and C53G SNPs.

Molecular docking-coupled dynamic simulations demonstrated significant binding for native *h*BD-2 being just lower than the reference *h*ACE2/spike RBD complex. The simulated native *h*BD-2 ligand showed overlapped binding at the spike RBD interface surface being similar to that for the reference *h*ACE2 complex. Binding to spike RBD is proposed to be residue-wise dependent since differential stability, fluctuation patterns, and binding energy contributions were assigned for each protein down to its amino acid level. Herein, we adopted the spike *Ser436*–*Tyr508* external loop domain for *h*BD-2 ligand anchoring as being significant for *h*ACE2 recognition and binding (5, 44, 55, 56). Reported studies showed preferential recognition of the spike RBD towards the *N*-terminal subdomain-1 of *h*ACE2 through predominant polar interactions (concentrated hydrogen bonding and salt bridges) between several hydrophilic residues along distinctive annealing regions (sites I and II) (74). Wang et al. showed that a hydrophobic stacking patch coexists for *Tyr489* and *Phe486* of RBD against Phe28, Leu79, Met82, and Tyr83 of *h*ACE2, yet does not greatly contribute to virus-receptor engagement since a single *L353A* mutation was sufficient to abolish such interactions (56). Therefore, polar anchoring of ligands at RBD/*h*ACE2 connective interface, with any of the interface polar residues, would probably impact both subdomain binding affinities or even alter the RBD conformation to be uncleavable *via* the host protease, TMPRSS2 (75, 76). Both suggested scenarios would halt the crucial stage of COVID-19 infection which is the virus–host membrane fusion and subsequent release of viral payload RNA into the host cytoplasm.

To our delight, both MM/GBSA and MM/PBSA binding energy calculations for respective docked and molecular dynamics complexes illustrated the predominance of electrostatic potentials and polar residue energy contributions for *h*BD-2 towards the spike RBD site. Greater negative values, suggesting stronger binding affinities, were consistent with the reported results of other research groups (5, 77). We decided to study the dimeric form of *h*BD-2, rather than their monomeric ones since these ligands exist at non-covalent hydrophobically bonded dimers within high concentrations (46, 78). Despite that, the reported *h*BD-2 dimerization could be quite modest and binding to spike RBD could stabilize the *h*BD-2 proteins at their oligomeric levels (5, 46). This was the case with the study by Zhang et al., where long ns all-atom simulation of dimeric *h*BD-2 showed higher free binding energies, greater hydrogen bond frequencies, and larger contact distances being maintained *via* both protomers as compared to the monomeric form (5).

Preferential binding of native *h*BD-2 over its SNP variants, being only second to the reference *h*ACE2/spike RBD complex, was demonstrated through a multi-level stability analysis. The RMSD analysis was significant for showing limited conformational changes and superior relative stability of the native *h*BD-2 complex depicting steady tones across the simulation times. On general bases, RMSD trajectories provide accurate measurement regarding a molecular deviation from its reference structure at the beginning of the molecular dynamics simulations (79). High-protein RMSDs usually correlate to significant conformation alterations and instability, while for ligands, they confer compromised ligand-target affinity and ligand-pocket accommodation (80). Both Rgs and BSAs were translated well for the RMSD findings, since these parameters depicted inherited stability, compactness, and larger contact distances for the native *h*BD-2 complex in relation to its SNP proteins. Generally, lower Rg values with limited fluctuations suggested optimum structural compactness in terms of favored inter- or intra-molecular interactions (81). In contrast, larger BSA across the simulation timeline is correlated with a bigger protein–protein contact interface denoted as the buried surface area between both molecules (82).

It is worth noting that the here-simulated *h*ACE2 and *h*BD-2 complexes were in dynamic motion at the spike RBD interface, which was consistent with the reported thermodynamic behavior of various protein–protein complexes (83–85). However, *h*BD-2 was more dynamic than *h*ACE2, which was suggested to furnish less unfavored entropy on binding than those obtained with complexes where both or either one partner is significantly rigid (5, 86). This was consistent with our MM/PBSA energy term contribution findings where *h*BD-2 complexes were of lower polar solvation entropy, which would favor ligand binding since the latter is considered a solvent-displacement process (87). Moreover, flexible peptide-based blocking strategies were suggested as beneficial to circumvent mutations that could compromise *h*BD-2 blocking affinities (88). Nevertheless, thermodynamic flexibility could be double-bladed since higher conformational changes seen with SNP proteins compared to the native form were suggested with ligand drifting. Additionally, depicted SNP dynamic behaviors were likely accompanied by indirect hydrogen bonding with water molecules at or even near the interface bridging such polar interactions, which would underestimate the electrostatic binding potential and further increase polar solvation entropic penalties resulting from displacing highly ordered water molecules at interacting protein surfaces. The latter was seen with several protein–protein complexes where one partner had more solvent exposure (22, 24, 89).

Notably, SNP mutant residues at *h*BD-2 were proposed to influence ligand binding and conformation, which was confirmed here through lower per-residue free binding energy contributions, reduced contact distance occupancies, and/or significant steric clashes with congruent protein interface being in concordance with current literature data (90–92). Computational analysis through molecular docking and dynamics studies revealed the detrimental impact of SNP at *h*BD-1 gene on the stability and protein function to bind with the bacterial phosphatidylinositol-4,5-bisphosphate (PIP-2) protein target. Some of the depicted SNPs were associated with a PIP-2 binding interface and compromised binding energies (93). Another study by Teng et al. evaluated the binding free energies of more than 260 protein–protein complexes with known SNPs *via* CHARMM forcefield/continuum electrostatic calculations where they revealed that these SNPs tend to destabilize the binding energy electrostatic components (94). Additionally, a study by Ranjith-Kumar et al. investigating the impact of SNP on Toll-like receptor-3 structure, expression, and function showed through molecular modeling that two relevant SNPs would alter the receptor conformation, particularly its leucine-rich repeated motifs, which was also correlated with defective receptor activation activity (95).

The engagement with gene–gene interactions became very important when investigating the disease–gene relationship as it was confirmed that various genetic loci show interactions between them (96). Using GeneMANIA showed that the *DEFB103A* gene and *CCR6* gene showed the closest connection with the *DEFB4A* gene. These genes with a close connection to *DEFB4A* may be affected by *DEFB4A* damaging SNPs as well.

Overall, our analysis showed the existence of three damaging missense mutations in *h*BD-2. These SNPs were predicted to affect the stability and the structure of *h*BD-2. Moreover, G51D and C53G mutations were located in highly conserved positions and had effects on the secondary structures of hBD-2. Furthermore, all-atom molecular dynamics simulations and free binding energy calculations assured that the native form has a preferential *h*BD-2 binding with the SARS-CoV-2 spike interface over the SNP mutant forms. Our results could increase our understanding of the genetic factors associated with COVID-19, which could improve the prevention as well as the management guidelines of such a serious disease (97, 98).

## Conclusion

The revealed role of *h*BD-2 in fighting against SARS-CoV-2 raised questions about the effects of the damaging missense mutations on this protective role against COVID-19. Our comprehensive investigation showed that three mutations in *h*BD-2 have the most damaging impact: P44L, G51D, and C53G SNPs. These SNPs showed decreasing effects on the stability of *h*BD-2 and damaging effects on the structure of *h*BD-2. G51D and C53G SNPs also had high conserved positions and showed alterations in *h*BD-2 secondary structures. Furthermore, our computational model showed preferential *h*BD-2 binding, for the native over the SNPs mutant forms, at site I and site II of the SARS-CoV-2 spike interface guided by predominant electrostatic binding potentials. This was confirmed by all-atom molecular dynamics simulations and free binding energy calculations. The further implementation of experimental procedures could pave the way for the identification of patients prone to COVID-19 and the development of new diagnostics and procedures for the management of this serious infectious disease.

## Data availability statement

The original contributions presented in the study are included in the article/**Supplementary Material**. Further inquiries can be directed to the corresponding authors.

## Author contributions

MYB, MAS, MAE, and KMD: conceptualization, methodology, and original draft preparation. HO, MSAZ, SA, GA, IJ, EF, MaA and SA: writing—review and editing. SSE, MA-D, JA and RAE: supervision and project administration. All authors contributed to the article and approved the submitted version.
