# Supplementary material for: HBD-2 variants and SARS-CoV-2: New insights into inter-individual susceptibility
Source: Front Immunol. 2022 Dec 9;13:1008463. doi: 10.3389/fimmu.2022.1008463 (PMC9780532; doi:10.3389/fimmu.2022.1008463)
Supplement: Supplementary file 1 [file DataSheet_1.docx]

**HBD-2 variants and SARS-CoV-2:**  **New insights into inter-individual susceptibility**

**Supplementary figures**

**
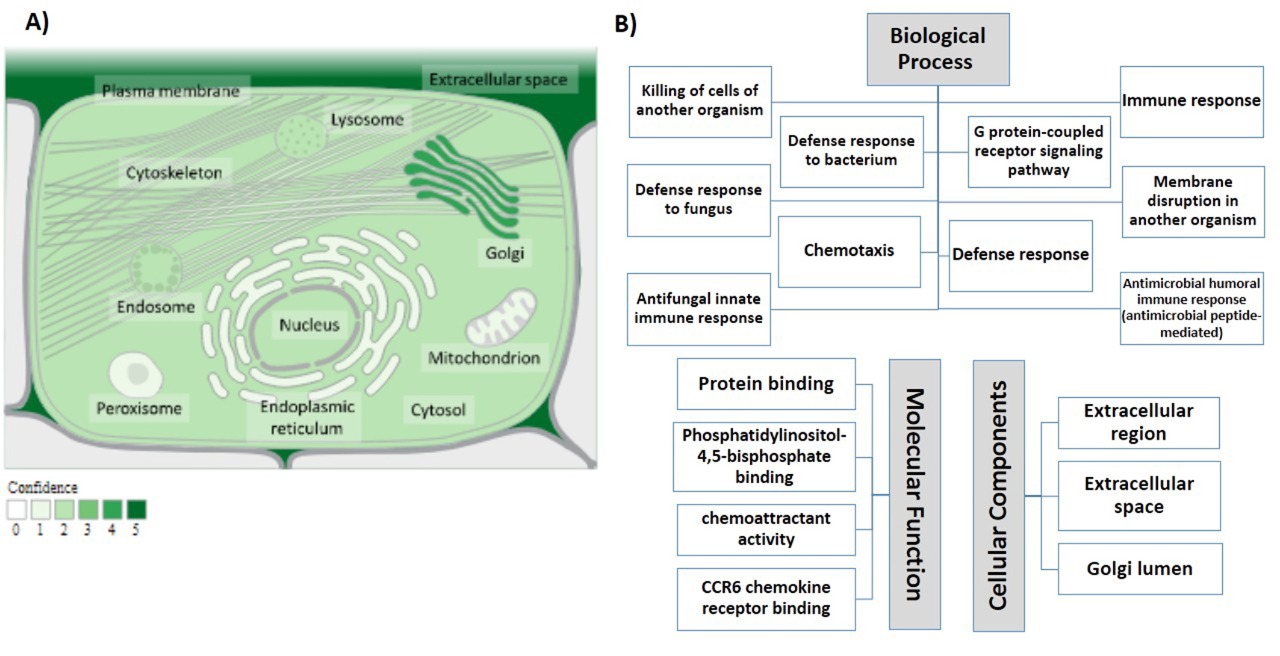
**

**Fig S1. S1A.** Subcellular localization related to *DEFB4A* gene, The levels of confidence are displayed in color code using a gradient where light green means low confidence and dark green which means a great level of confidence (genecards.org/) with (Compartments.jensenlab.org/) represents the image source. **S2A. Analysis of the gene ontology of *DEFB4A* gene.** Terms of molecular function, cellular componenets and biological process are displayed (source of data: genecards.org).

**
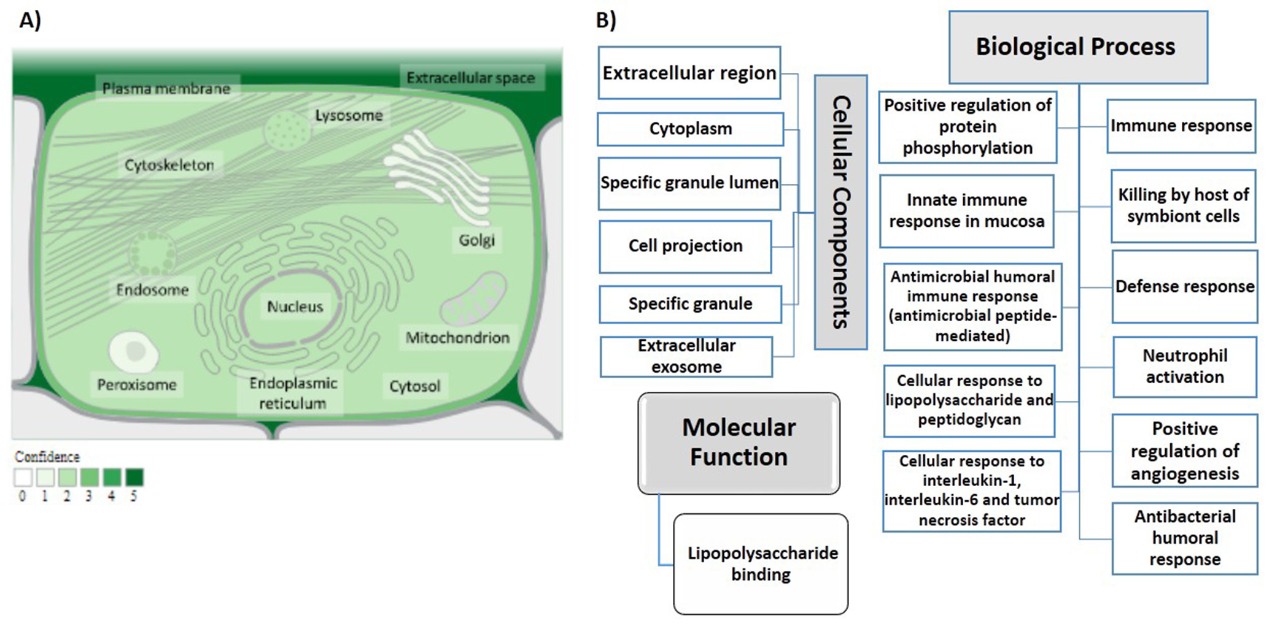
**

**Fig S2. S1A. Subcellular localization related to *CAMP* gene.** The levels of confidence are displayed in color code using a gradient where light green means low confidence and dark green which means a great level of confidence (genecards.org/) with (Compartments.jensenlab.org/) represents the image source. **S2A. Analysis of the gene ontology of *CAMP* gene.** Terms of molecular function, cellular componenets and biological process are displayed (source of data: genecards.org).

**
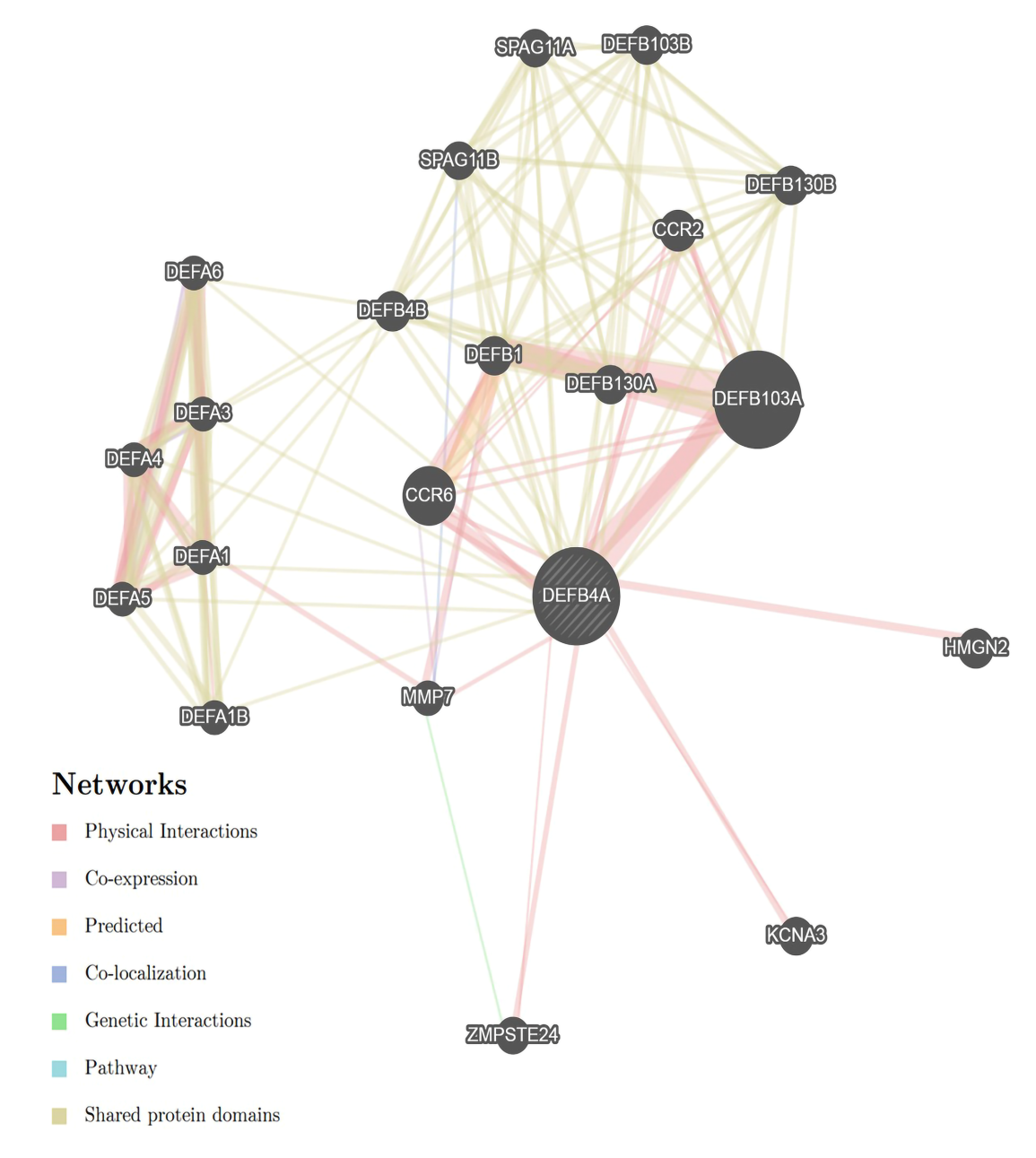
**

**FigS3. *DEFB4A g*ene-gene interaction analysis as created by GeneMANIA.**

**Supplementary tables**

**Table S1. Prediction and scores of deleterious missense SNPs by six in silico tools in LL-37.**

|  | SNP Id | AA change | SIFT | | PolyPhen-2 | | PROVEAN | | SNP&GO | | PHD-SNP | | SNAP2 | |
| --- | --- | --- | --- | --- | --- | --- | --- | --- | --- | --- | --- | --- | --- | --- |
|  |  |  | Prediction | score | Prediction | score | Prediction | score | Prediction | RI score | Prediction | RI score | Prediction | score |
| 1 | rs2033493828 | G136S | TOLERATED | 0.09 | BENIGN | 0.300 | Neutral | -0.537 | Neutral | 9 | Neutral | 7 | effect | 36 |
| 2 | rs199533593 | G136A | TOLERATED | 0.29 | BENIGN | 0.036 | Neutral | -0.617 | Neutral | 10 | Neutral | 9 | neutral | -2 |
| 3 | rs2033493930 | D137V | Deleterious low confidence | 0.05 | BENIGN | 0.038 | Neutral | -1.54 | Neutral | 9 | Neutral | 6 | effect | 35 |
| 4 | rs560486798 | F138L | TOLERATED | 0.85 | BENIGN | 0.002 | Neutral | -0.579 | Neutral | 9 | Neutral | 9 | effect | 33 |
| 5 | rs141472233 | R140W | Deleterious low confidence | 0 | BENIGN | 0.046 | Deleterious | -2.568 | Neutral | 8 | Neutral | 3 | effect | 71 |
| 6 | rs531701238 | R140Q | TOLERATED | 0.28 | BENIGN | 0.020 | Neutral | -1.129 | Neutral | 9 | Neutral | 9 | effect | 34 |
|  |  | R140P | Deleterious low confidence | 0.02 | POSSIBLY DAMAGING | 0.866 | Neutral | -1.92 | Neutral | 7 | Disease | 4 | effect | 67 |
| 7 | rs974064219 | K143E | TOLERATED | 0.29 | BENIGN | 0.016 | Neutral | -0.873 | Neutral | 10 | Neutral | 3 | effect | 45 |
| 8 | rs780840779 | E144G | Deleterious low confidence | 0.03 | BENIGN | 0.092 | Neutral | -1.549 | Neutral | 9 | Neutral | 6 | effect | 61 |
| 9 | rs2033494375 | K145Q | Deleterious low confidence | 0.01 | BENIGN | 0.441 | Neutral | -1.421 | Neutral | 8 | Neutral | 6 | effect | 39 |
| 10 | rs55708841 | K145R | TOLERATED | 0.08 | POSSIBLY DAMAGING | 0.528 | Neutral | -0.516 | Neutral | 9 | Neutral | 7 | neutral | -10 |
| 11 | rs771329723 | I146F | Deleterious low confidence | 0.03 | BENIGN | 0.067 | Neutral | -0.513 | Neutral | 9 | Neutral | 5 | effect | 40 |
| 12 | rs1331560117 | I146T | Deleterious low confidence | 0 | POSSIBLY DAMAGING | 0.856 | Neutral | -2.015 | Neutral | 7 | Neutral | 1 | effect | 66 |
| 13 | rs1487865661 | F150L | TOLERATED | 1 | BENIGN | 0.000 | Neutral | 0.937 | Neutral | 10 | Neutral | 9 | effect | 19 |
| 14 | rs760073514 | R152I | Deleterious low confidence | 0.01 | BENIGN | 0.007 | Deleterious | -2.564 | Neutral | 10 | Neutral | 8 | effect | 40 |
| 15 | rs2033495004 | I153T | Deleterious low confidence | 0 | BENIGN | 0.006 | Neutral | -1.625 | Neutral | 6 | Neutral | 4 | effect | 47 |
| 16 | rs2033495087 | R156G | Deleterious low confidence | 0.01 | BENIGN | 0.001 | Neutral | -1.956 | Neutral | 8 | Neutral | 5 | effect | 42 |
| 17 | rs530715339 | R156K | TOLERATED | 1 | BENIGN | 0.001 | Neutral | 0.339 | Neutral | 10 | Neutral | 10 | neutral | -1 |
| 18 | rs374668266 | R162W | Deleterious low confidence | 0.03 | POSSIBLY DAMAGING | 0.448 | Neutral | -1.93 | Neutral | 8 | Neutral | 8 | effect | 64 |
| 19 | rs760861316 | R162Q | TOLERATED | 0.47 | BENIGN | 0.002 | Neutral | 0.167 | Neutral | 10 | Neutral | 9 | effect | 29 |
| 20 | rs764354350 | N163H | Deleterious low confidence | 0.04 | POSSIBLY DAMAGING | 0.621 | Neutral | -0.689 | Neutral | 9 | Neutral | 8 | effect | 28 |
|  |  | N163Y | Deleterious low confidence | 0.02 | BENIGN | 0.031 | Neutral | -1.344 | Neutral | 9 | Neutral | 2 | effect | 32 |
| 21 | rs762011878 | L164P | Deleterious low confidence | 0 | BENIGN | 0.012 | Neutral | -0.726 | Neutral | 9 | Disease | 3 | effect | 63 |
|  |  | L164R | Deleterious low confidence | 0 | BENIGN | 0.360 | Neutral | -1.509 | Neutral | 6 | Disease | 4 | effect | 70 |
| 22 | rs2033495676 | V165I | TOLERATED | 0.2 | BENIGN | 0.002 | Neutral | -0.157 | Neutral | 10 | Neutral | 7 | effect | 1 |
| 23 | rs1427760452 | P166H | Deleterious low confidence | 0 | PROBABLY DAMAGING | 0.927 | Deleterious | -2.909 | Neutral | 7 | Neutral | 4 | effect | 58 |
| 24 | rs1414856090 | R167K | TOLERATED | 0.05 | BENIGN | 0.098 | Neutral | -0.821 | Neutral | 9 | Neutral | 9 | effect | 46 |
| 25 | rs1441070588 | T168A | TOLERATED | 0.07 | BENIGN | 0.026 | Neutral | -0.422 | Neutral | 10 | Neutral | 8 | effect | 9 |
| 26 | rs765071419 | T168I | Deleterious low confidence | 0.02 | BENIGN | 0.004 | Neutral | -0.224 | Neutral | 10 | Neutral | 4 | effect | 19 |
| 27 | rs1470005902 | S170F | Deleterious low confidence | 0 | BENIGN | 0.142 | Neutral | -0.393 | Neutral | 10 | Neutral | 8 | effect | 31 |
